# Cellular respiration and amino acid metabolism is altered by dietary oligosaccharides in *Salmonella* with epithelial cell association

**DOI:** 10.3389/fmicb.2025.1672770

**Published:** 2025-10-07

**Authors:** Claire A. Shaw, Poyin Chen, Narine Arabyan, Bart C. Weimer

**Affiliations:** Population Health and Reproduction, 100K Pathogen Genome Project, School of Veterinary Medicine, Davis, CA, United States

**Keywords:** non-typhoidal infection, prebiotics, host association, infectious disease, nitrogen metabolism, sugar metabolism

## Abstract

**Introduction:**

Dietary prebiotic oligosaccharides, complex carbohydrates that support beneficial bacteria, are ubiquitous on marketplace shelves and in people’s diets. Though widely accessible and consumed, little is known about how different prebiotics alter the epithelium and microbes during enteric infections.

**Methods:**

Here we show two structurally different prebiotic oligosaccharides, human milk oligosaccharides (HMO) and mannanoligosaccharides (MOS), alter the metabolism of colonic epithelial cells and *Salmonella enterica* sv. Typhimurium in ways specific to each prebiotic during infection in a focused ‘*in vitro*’ model.

**Results:**

Initially, HMO and MOS addition decreased *S*. Typhimurium association with epithelial cells. However, gene expression analysis revealed significantly induced expression of Specific Pathogenicity Island (SPI) 1 (adj. *p* < 2.0−6) and 2 (adj. *p* < 3.0−5) with HMO treatment, opposed to increased fimbriae expression (adj.*p* < 3.0−3) with MOS treatment. Both host and pathogen metabolism were likewise altered with prebiotic addition. MOS treatment induced the expression of genes for amino acid metabolism in both the host cells and in *S*. Typhimurium, a metabolic shift that was not observed in the HMO treated cells. MOS treatment also altered pathogen-related respiration metabolism in *S*. Typhimurium toward activity typically seen during gut inflammation.

**Discussion:**

The regulation of virulence expression in *Salmonella* from prebiotic treatment was unexpected and suggests prebiotics act in context-dependent ways to potentiate or attenuate enteric activity.

## Introduction

Dietary additives that are reported to selectively feed beneficial gut bacteria, termed prebiotics, are commonplace and advertised to have a myriad of health benefits. The US prebiotic market alone currently exceeds $6 Billion and is growing rapidly (Mano et al., 2018). Prebiotic oligosaccharides, complex carbohydrates consisting of between three and 10 monosaccharides, are one prebiotic grouping thought to provide beneficial health effects through their roles as metabolic substrates for probiotic bacteria (Maathuis et al., 2012; Goh and Klaenhammer, 2015) and through modulation of the intestinal barrier (Rentas et al., 2020; Commane et al., 2005; Perdijk et al., 2019). Though classified together under a single label, prebiotic oligosaccharides are structurally diverse and differ in their overall monosaccharide composition. Fructooligosaccharides (FOS), galactooligosaccharides (GOS), and mannanoligosaccharides (MOS) are all commercially available functional oligosaccharides derived from different source material (Cheon et al., 2023; Tingirikari, 2018). Not yet commercialized for broad consumption are human milk oligosaccharides (HMO). HMOs are a combination of structurally diverse oligosaccharides produced by the mother in breast milk, which subsequently function as a selective bacterial carbon source in the large intestine of infants (Plaza-Diaz et al., 2018). This structural and functional range of prebiotic oligosaccharides increases the complexity around disentangling substrate-microbe-host interactions and their related health outcomes (You et al., 2022). Prebiotic research has primarily focused on the beneficial aspects of prebiotics and their use in combination with probiotic bacteria (Liu et al., 2019; Hibberd et al., 2019). In contrast, relatively little research has focused on the impact of prebiotics on important enteric pathogens, including *Salmonella enterica* sv. Typhimurium.

Previous studies have used prebiotics for the *in vitro* and *in vivo* control of enteric pathogens such as *Escherichia coli*, *Listeria monocytogenes* and *Salmonella enterica* sv. Enteritidis with limited success (Shoaf et al., 2006; Chen et al., 2017; Newman and Arshad, 2020; Velez et al., 2013; Khan et al., 2020). Certain prebiotics like HMOs and GOS contain glycan structures similar to those found on the gut epithelial cell surface and used by pathogens for host adherence (Bode, 2012), promoting pathogen-prebiotic binding and simulateanosulty decreasing pathogen-host interactions. Enteropathogenic *E. coli* incubated with GOS prior to host introduction showed significantly decreased host association (Shoaf et al., 2006). However, GOS was unable to displace already adhered *E. coli*, suggesting prebiotic-pathogen binding prevented initial adherence to host cells (Shoaf et al., 2006). Similar decoy mechanisms have been shown with *Campylobacter jejuni* and α -1-2-fucosylated glycans, a component of HMO (Ruiz-Palacios et al., 2003). Though *S.* Typhimurium is the most prevalent enteric pathogen in humans and is responsible for over 80 million cases of foodborne illness and 155,000 deaths per year globally (Gong et al., 2022), relatively little is known about the interaction of *S.* Typhimurium and prebiotic substrates during infection.

Utilizing a focused system to examine differentiated Caco2 cells during infection with *S.* Typhimurium without any additional microbiota, we showed two structurally different prebiotic oligosaccharides, HMO and commercially available MOS (BioMos^®^) differentially drive host-pathogen metabolic crosstalk in ways unique to each prebiotic. Mannan-oligosaccharides (MOS) are yeast-derived prebiotics composed of mannose residues, while HMOs are structurally diverse glycans naturally present in breast milk, with both suggested to have pathogen-regulating potential (Spring et al., 2015; Craft and Townsend, 2018). Studying both provides insight into how distinct oligosaccharide structures shape host–pathogen dynamics, particularly in relation to *Salmonella* virulence and colonization. We specifically observed differences in amino acid metabolism and two major energy-producing routes that altered redox balance in the host-pathogen system and modulated host-pathogen metabolic interactions. Though *S.* Typhimurium virulence factor expression was induced, pathogen-host association was attenuated by both prebiotic treatments, indicating that a complex relationship exists between prebiotics and gut pathogens. The context-dependent and substrate-specific prebiotic effects observed here necessitate further mechanistic research into the ameliorative or pathogen potentiating properties of dietary substrates.

## Results

### Prebiotic pre-treatment of Caco2 cells decreases Salmonella association

**Figure 1:**
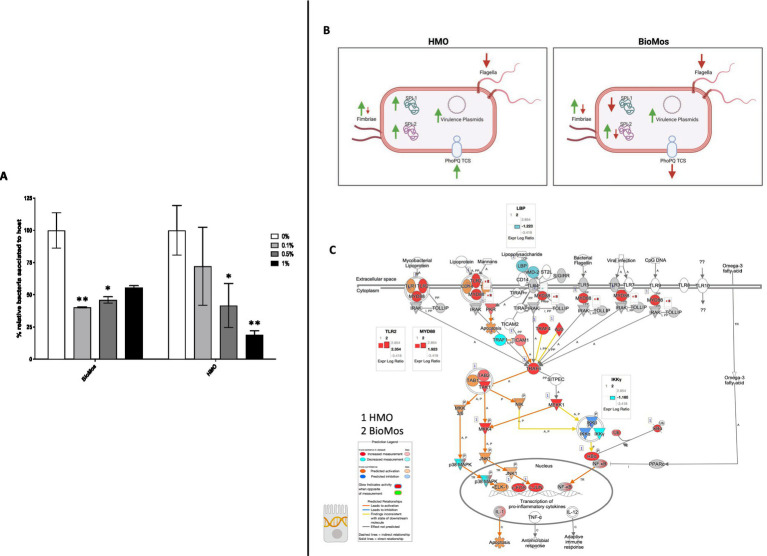
Adhesion and invasion activity of *Salmonella Typhimurium* in response to prebiotic pre-treatment. **(A)** Prebiotic pre-treatment of Caco2 cells reduces combined invasion and adhesion of Salmonella LT2 in an in-vitro setting. 0% prebiotic addition (white bars) are used as the control for comparison of increasing (0.1, 0.5, and 1%) prebiotic treatment concentrations. BioMos and HMO were added at the noted concentrations to differentiated Caco2 cells for 15 min, followed by co-incubation of *S.* Typhimurium LT2 for 60 min. Invasion and adhesion was measured with a gentamicin protection assay and % associated bacteria values represent the combined percentage of Salmonella cells which invaded or adhered to host cells. *p < 0.05, **p < 0.02. **(B)** Expression of virulence factors in *S.* Typhimurium 14028 ss was evaluated for each prebiotic treatment, using *S.* Typhimurium 14028 ss without prebiotics as the control. Green up arrows represent an upregulation of genes related to each factor while red down arrows represent repression. **(C)** The TLR expression pathway for Caco2 cells was overlayed with both HMO and BioMos treated Caco2 expression profiles, with no prebiotic Caco2s as the control for both cases. Teal represents decreased measurement for the gene while red represents increased measurement. Blue and orange genes represent predicted inhibition or activation, respectively. Inset graphs display the log2 fold-change comparison between HMO (1) and BioMos (2).

Pre-treatment of Caco2 cells with structurally different oligosaccharides altered the combined invasion and adhesion activity of *S. enterica* serovar Typhimurium LT2 in a dose-dependent manner (Figure 1). Pre-treatment of the Caco2 cells at all tested concentrations with either BioMos^®^ or HMO reduced the association of *S.* Typhimurium LT2 with the differentiated host cells. When added at 0.1% (w/v), BioMos^®^ showed a 59% reduction (*p* < 0.02) in adhesion and invasion while HMO showed a lesser effect at 28% association reduction (non-significant). At 0.5% BioMos^®^ reduced *S.* Typhimurium LT2 association by 54% (*p* < 0.05) and HMO by 59% (*p* < 0.05). The maximum tested concentration, 1% prebiotic, had a 44% reduction (non-significant) in association in the BioMos^®^ treatment while at this same concentration, HMO addition resulted in an 82% decrease in *S.* Typhimurium LT2 association (*p* < 0.02). Increased levels of BioMos^®^ decreased efficacy of the pre-treatment while increased HMO levels increased efficacy.

### Expression of *Salmonella* virulence factors and Caco2 receptors altered by prebiotics

*Salmonella typhimurium* 14028 s is 98% genetically identical to *S.* Typhimurium LT2 (Wahlig et al., 2019), so the dose dependent response to prebiotic treatment seen in LT2 was predicted to carry over to *S.* Typhimurium 14028 s activity. *S.* Typhimurium 14028 s, being more pathogenic than the lab strain LT2 and thus more relevant to *in vivo* activity, was used to evaluate the response of host-pathogen interactions to prebiotic treatment via metatranscriptomics and metabolomics.

**Figure 2:**
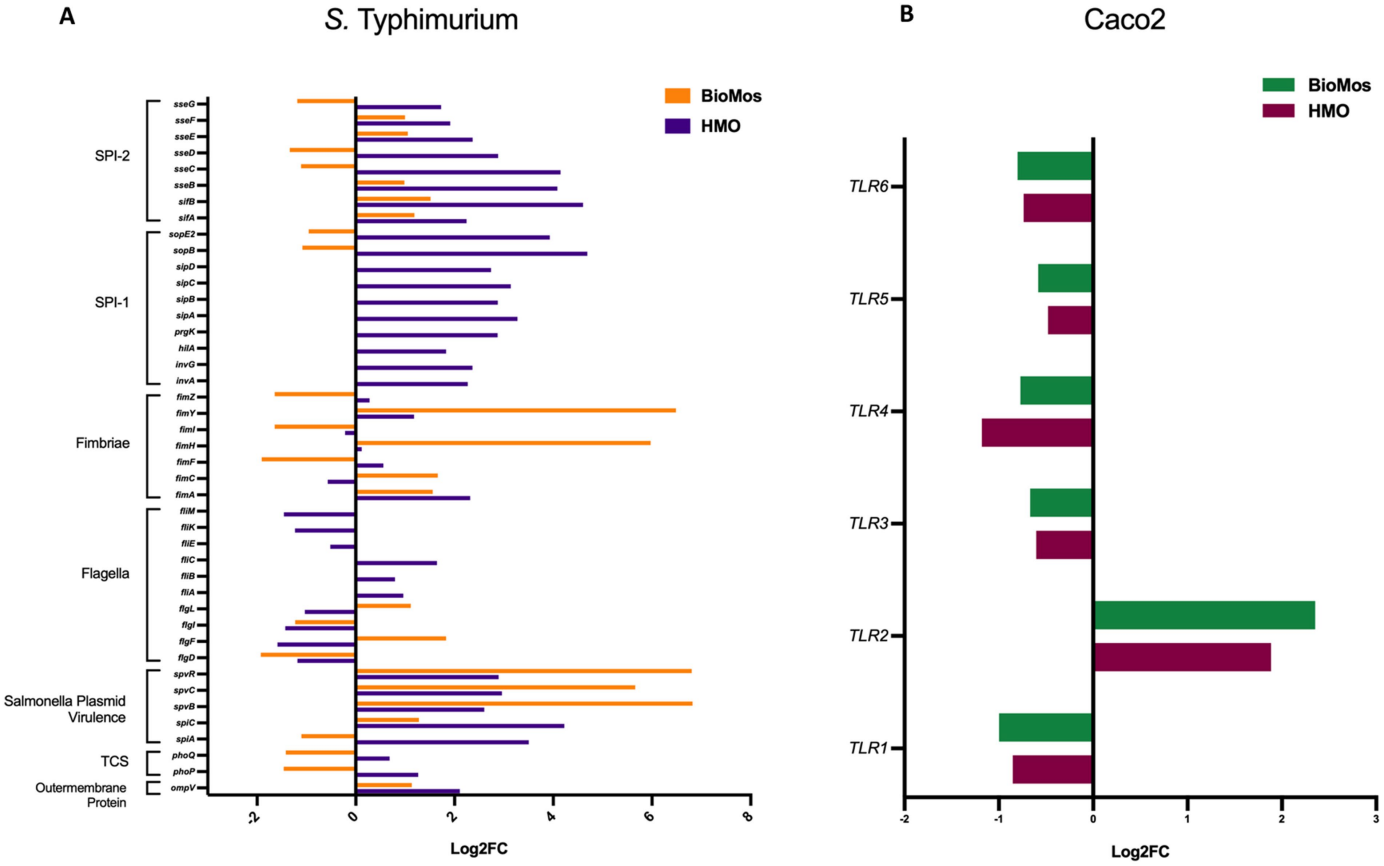
Expression of virulence factors in *S.* Typhimurium 14028 ss and TLRs in Caco2 Cells. **(A)** Virulence factor expression in *S.* Typhimurium added to prebiotic treated Caco2 cells was measured as log_2_ fold change from *S.* Typhimurium exposed to untreated Caco2 cells after 60 min of co-incubation. Orange represents *S.* Typhimurium add to the BioMos condition and purple represent the HMO condition *S.* Typhimurium. **(B)** Toll-like receptor expression in prebiotic treated then infected Caco2 cells was evaluated by log_2_ fold change expression data with non-treated but infected Caco2 cells as control. Green represents BioMos treatment and purple represents HMO treatment.

The reduction in association to host cells of *Salmonella* in both prebiotic conditions led us to investigate if the modulation of known virulence factors in *S.* Typhimurium 14028 s and pathogen-sensing Toll-Like Receptors (TLRs) in the Caco2 cells contributed to the defects in adhesion and invasion. Salmonella Pathogenicity Island 1 (SPI-1) and Salmonella Pathogenicity Island 2 (SPI-2) are important gene cassettes that drive *Salmonella* virulence, with SPI-1 supporting initial adhesion to and invasion of host cells and SPI-2 aiding in vacuole escape and systemic spread (Hume et al., 2017). Thirty-four out of the 43 (79.1%) virulence genes detected in this analysis were induced in the HMO condition, while only 14 of the 43 (32.6%) were induced by BioMos^®^ treatment. All genes related to SPI-1 and SPI-2 were significantly induced (between −log_10_*P* = 6.7 and 17.6, adj. *p* < 3.0e-5) in the HMO treatment while multiple SPI-1 and SPI-2 genes were repressed BioMos^®^ treatment (Figure 1B). SPI-2 genes *sseG, sseD,* and *sseC* were repressed and *sseF, sseE, sseB, sifB,* and *sifA* were induced with BioMos^®^ addition (Figure 2), suggesting invasion into the epithelial cell was unchanged.

Fimbriae, structures important for adhesion prior to invasion (Kolenda et al., 2019), likewise displayed mixed expression patterns between the prebiotic treatments. *Salmonella Typhimurium* 14028 s combined with HMO treated Caco2 cells showed induction of *fimA, fimF, fimY,* and *fimZ*. Whereas *S.* Typhimurium 14028 s combined with BioMos^®^ treated Caco2 cells repressed *fimF, fimI,* and *fimZ,* but significantly induced *fimY* (log_2_FC = 6.5, −log_10_*P* = 11.5), which encodes a mannose-binding type one fimbriae (Wang et al., 2014). Expression of virulence genes is in part controlled by two-component systems (TCS). Given their role in regulating virulence, the expression of 13 TCSs across prebiotic treatments was evaluated (Table 1). Generally, TCS-related transcripts in *S.* Typhimurium 14028 s under the BioMos^®^ treatment were either not found or were repressed. In contrast, HMO treated *S.* Typhimurium 14028 s showed differential expression of all 13 TCSs. Most were repressed compared to expression in *S.* Typhimurium 14028 s without HMO, though the expression of stress-response TCS, *rpoS*/*rssB* (Rychlik and Barrow, 2005), was induced, as was the *arcA* receptor from *arcA/arcB* TCS.

**Table 1:** Expression of two-component systems by *S.* Typhimurium, broken down by sensor and regulator expression.

TCS pair	Component	Gene ID	HMO (Log_2_FC)	HMO (−Log_10_*P*)	BioMos (Log_2_FC)	BioMos (−Log_10_*P*)
ArcB/ArcA	Sensor	*STM14_RS17695*	−0.82	2.10		
	Regulator	*STM14_RS17695*	2.37	12.54		
BaeS/BaeR	Sensor	*STM14_RS22535*	−1.27	3.49		
	Regulator	*STM14_RS22540*	−0.50	1.20		
BarA/UvrY	Sensor	*STM14_RS15835*	−0.53	1.37		
	Regulator	*STM14_RS10645*	1.38	4.27		
CpxA/CpxR	Sensor	*STM14_RS21360*	−1.30	2.51		
	Regulator	*STM14_RS21365*	−0.56	1.19		
CreC/CreB	Sensor	*STM14_RS24035*	−1.03	2.16		
	Regulator	*STM14_RS24030*	−0.74	1.37		
DcuS/DcuR	Sensor	*STM14_RS22600*	−0.04	0.06		
	Regulator	*STM14_RS22595*	0.92	2.38		
EnvZ/OmpR	Sensor	*STM14_RS18570*	−1.07	2.79		
	Regulator	*STM14_RS18575*				
GlrK/GlrR	Sensor	*STM14_RS14045*	−0.91	3.09		
	Regulator	*STM14_RS14035*	−0.93	2.05		
KdbD/KdpE	Sensor	*STM14_RS04130*	−1.10	1.42	−1.77	3.76
	Regulator	*STM14_RS04125*	−0.98	2.07	−1.82	3.76
NarX/NarL	Sensor	*STM14_RS09720*	−0.58	1.20	0.85	1.76
	Regulator	*STM14_RS09725*	−0.15	0.21	1.40	1.76
PhoQ/PhoP	Sensor	*STM14_RS06660*	0.69	1.79	−1.42	2.62
	Regulator	*STM14_RS06665*	1.26	3.95	−1.46	2.61
RstB/RstA	Sensor	*STM14_RS08210*	−1.07	2.76	−1.39	2.17
	Regulator	*STM14_RS08230*	0.42	0.52	−1.11	2.16
TtrS/TtrR	Sensor	*STM14_RS07785*	−1.10	2.33	−1.21	2.28
	Regulator	*STM14_RS07790*	−0.51	0.87	−1.49	2.28

The expression patterns of virulence factors alone did not support the observed prebiotic-driven reduction in host association. We expanded our analyses to include expression of glycosyl hydrolases, which are *Salmonella* enzymes needed to access the host surface and are suspected to be among emerging virulence factors that enable luminal pathogens to achieve contact with the host cell membrane (Arabyan et al., 2016) prior to involvement of the T3SS. Glycosyl hydrolases of *S.* Typhimurium under the HMO condition were repressed - *bcsC, bglX, nagZ, malS, nanH,* and *mltB* (Table 2). Of the aforementioned genes, BioMos^®^ treated *S.* Typhimurium repressed *nagZ* (Log_2_FC = −1.44, −log_10_*P* = 2.6) and induced *glgX* (Log_2_FC = 0.956, −log_10_*P* = 2.1), and *glgB* (Log_2_FC = 1.04, −log_10_*P* = 2.1) expression. These combined observations suggest that HMO treatment led to repression of multiple glycan degrading enzymes needed to access plasma membrane, thereby hindering bacterial accessibility to the plasma membrane and subsequent host invasion.

**Table 2:** Expression of glycosyl hydrolase enzymes by *S.* Typhimurium.

Common gene name	Gene ID	HMO (Log_2_FC)	HMO (−Log_10_*P*)	BioMos (Log_2_FC)	BioMos (−Log_10_*P*)	Characterization
*bcsC*	STM14_RS19140	−1.54	3.2			Endoglucanase
*bglX*	STM14_RS12040	−1.52	4.8			Beta-glucosidase
*nagZ*	STM14_RS06550	−0.78	2.7	−1.44	2.6	Beta-hexosaminidase
*malS*	STM14_RS19390	−0.75	1.6			Alpha-amylase
*nanH*	STM0928	−0.74	1.3			Neuraminidase
*mltB*	STM14_RS15190	−0.10	0.2			Murein-transglycosylase B
*glgB*	STM14_RS08670	0.13	0.2	1.04	2.1	Malto-oligosyltrehalose trehalohydrolase
*bglA*	STM14_RS16310	0.78	2.6			Sialidase
*glgX*	STM14_RS08660	0.837	3.3	0.95	2.1	Glycogen debranching enzyme
STM0907	STM14_RS11050	0.83	1.5			Chitinase

**Figure 3:**
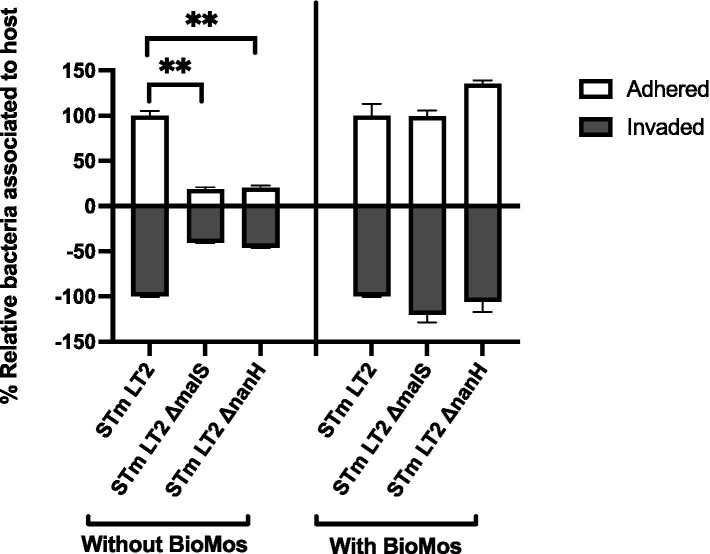
BioMos addition rescues invasion phenotype of *S.* Typhimurium LT2 glycosyl hydrolase knockouts. Single gene knockouts of an amylase encoding gene (malS) and sialidase encoding gene (nanH) decreased the ability of *S.* Typhimurium LT2 to both adhere to and invade Caco2 cells. Pretreatment of the Caco2 cells with 1% BioMos for 60 min restored the adhesion and invasion abilities of both the malS and nanH knockouts as compared to a wild-type control. *S.* Typhimurium was co-incubated with the Caco2 cells for 60 min and invasion and adhesion was measured with a gentamicin protection assay. *p < 0.05, **p < 0.02.

The importance of glycosyl hydrolase enzymes in supporting *S.* Typhimurium invasion of Caco2 cells was evaluated using *malS* and *nanH* knockouts, which encode for an amylase and sialidase, respectively, in the same prebiotic pre-treated Caco2 cell setup as used for Figure 1 and as described in previous work (Arabyan et al., 2016). As expected, deletion of *nanH* and *malS* reduced both the adhesion and invasion of *S.* Typhimurium LT2 to Caco2 cells (Figure 3), indicating these sialidase and amylase enzymes are necessary for pathogen access to the host cell membrane. Unexpectedly, BioMos^®^ rescued the invasive phenotype of these same glycosyl hydrolase knockouts (Figure 3).

### Host surface receptor expression differentiation with prebiotics

Prebiotic pretreatment of polarized Caco2 monolayers resulted in differential expression of multiple transmembrane receptors in a prebiotic-specific manner, but this differential regulation alone does not explain the difference in the association activity of *S.* Typhimurium 14028 s (Supplementary Figure 1). To better understand how prebiotics modulated the interaction of the host Caco2 cells and pathogen in this model, we looked at the expression of TLRs, which are integral receptors for initiating an immune response (Abreu et al., 2005). Both prebiotic pretreatments resulted in the same TLR expression pattern (Figure 2). *TLR2* was the only membrane receptor induced with prebiotic pretreatment, and previous work has shown the yeast component zymosan and the HMO structure 3-fucosylactose both induce host *TLR2* expression (Dillon et al., 2006; Cheng et al., 2019). *TLR1, 3, 4, 5,* and *6* were similarly repressed in both HMO and BioMos^®^ conditions. Expression of the integral TLR signaling protein, MYD88, was induced in both prebiotic conditions, indicating the Caco2 monolayer sensing of *S.* Typhimurium 14028 s in both treatments. These data indicate that host-prebiotic interactions through surface mediated contact regulates intracellular activity. We further investigated the influence of prebiotic-transmembrane receptor interactions on additional extrinsically modulated signaling cascades, i.e., metabolic activity.

### Prebiotic pre-treatment of Caco2 cells modulates the expression of metabolic pathways

**Figure 4:**
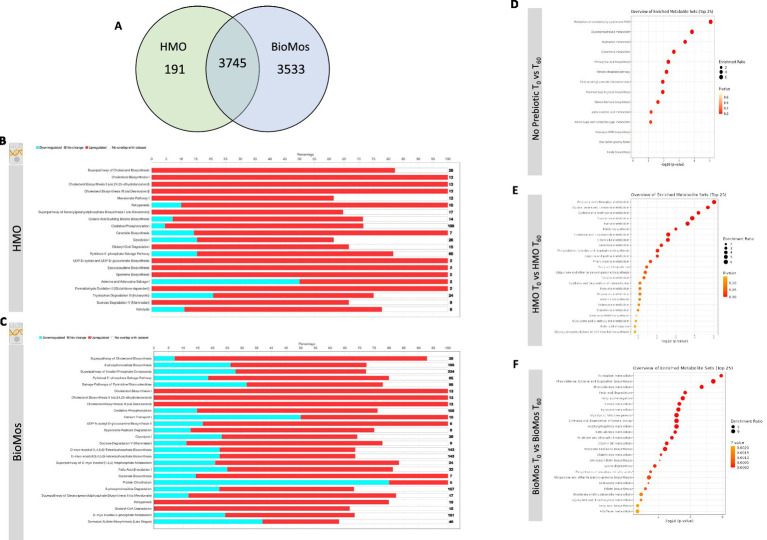
Regulation of gene expression and metabolism of Caco2 cells in response to prebiotic pretreatment. **(A)** HMO and BioMos treatment of Caco2 cells resulted in 3,745 shared significantly expressed genes, 191 additional unique genes in HMO treated cells and 3,533 in BioMos treated cells. **(B,C)** IPA was used to evaluated the expression patterns of the most significant (−log10*P* > 1.3) metabolic pathways in both prebiotic treatments. Bars represent the percentage of each pathway differentially expressed in each condition, red sections being upregulation, blue downregulation, and white as the percentage of genes found to be unsignificant in the treatment for that pathway. Right hand side numbers represent the total genes in each pathway. **(D–F)** The metabolic profiles of prebiotic treated Caco2 cells were analyzed using Metabolite Set Enrichment Analysis (MSEA) in MetaboAnalyst. Time 0 with no prebiotic **(D)**, HMO **(E)**, or BioMos **(F)** was used as the control comparison for the same condition at Time 60. MSEA graph display the enriched pathways at Time 60 for each prebiotic condition, with the dot size representing the portion of the pathway enriched in the dataset and color as *p*-value.

The incubation of Caco2 cells with prebiotics prior to infection altered the expression of metabolic pathways, as compared to Caco2 cells infected without any prebiotic pretreatment. Both HMO and BioMos^®^ significantly altered expression of the same 3,745 genes as compared to no prebiotic, but BioMos^®^ treatment significantly modulated expression of an additional 3,533 unique Caco2 genes compared to HMO treatment (−Log_10_ *p* > −1.3) (Figure 4). HMO treatment changed the expression of only 191 unique genes when compared to BioMos^®^. Both HMO and BioMos^®^ treated, then infected, Caco2 cells had significant enrichment of transcripts related to multiple cholesterol biosynthesis pathways, which are a known response to *S.* Typhimurium 14028 s infection (Goluszko and Nowicki, 2005), as well as the upregulation of genes related to ketogenesis and oxidative phosphorylation. Overall, HMO and BioMos^®^ treated and *S.* Typhimurium 14028 s infected Caco2 cells shared enriched expression of the same 11 metabolic pathways, while HMO treated cells had an addition 10 pathways and BioMos^®^ cells had 15 uniquely enriched pathways (Figure 4).

Caco2 cells pretreated with HMO and infected with *S.* Typhimurium 14028 s induced expression of epoxysqualene biosynthesis, spermine biosynthesis, and mevalonate pathway I, which are all involved in cholesterol biosynthesis and regulation. Genes related to broad level lipid metabolism are induced in the presence of HMO amid an ongoing infection with *S.* Typhimurium 14028 s. The differential regulation of spermine, a polyamine derived from arginine and ornithine, in the presence of HMO and *Salmonella* challenge is particularly notable as the production of polyamines by the host has been previously shown to fuel pathogenic activity in *S.* Typhimurium (Catron et al., 2004; Jelsbak et al., 2014). Additionally, the tryptophan degradation was modulated by HMO treatment but was not significantly altered in BioMos^®^ treated cells. In HMO treated cells, 20% of genes for the tryptophan degradation pathway were repressed, 57% were induced, and 23% were not found in the data. In contrast to the general induction of metabolic pathways seen in HMO treated Caco2s, BioMos^®^ treated cells displayed more mixed regulation, with the majority of significant pathways displaying a combination of repressed and induced genes. Notably the lipid-related ceramide biosynthesis and protein citrullination pathways have significantly altered expression in BioMos^®^ treated cells, though ceramide biosynthesis is primarily induced while protein citrullination was repressed.

Pathway enrichment analysis for metabolites from the control, HMO, and BioMos^®^ treatments compared across 60 min of prebiotic incubation with polarized Caco2 cells revealed both prebiotic treatments resulted in a greater number of significantly (adj. *p* < 0.05) enriched pathways in Caco2 cells as compared to Caco2 cells without any prebiotic addition (Figure 4). Absent prebiotic pretreatment, metabolism of xenobiotics by cytochrome P450 as the most significantly enriched pathway (−Log_10_ *p* > 1.3), followed by glycerophospholipid metabolism, tryptophan metabolism, glutathione metabolism and primary bile acid biosynthesis. Both prebiotic metabolic enrichment comparisons also had glutathione metabolism and tryptophan metabolism as an enriched pathway after 60 min of prebiotic pretreatment/infection (Figure 4). The top five pathways for HMO treated Caco2 cells were porphyrin and chlorophyll metabolism, glycine, serine, and threonine metabolism, cysteine and methionine metabolism, tryptophan metabolism, and purine metabolism. In BioMos^®^ treated Caco2 cells the top five pathways were tryptophan metabolism, phenylalanine, tyrosine, and tryptophan biosynthesis, phenylalanine metabolism, fatty acid degradation, and fatty acid elongation. Notably both prebiotic treatments resulted in the enrichment of multiple pathways related to amino acid metabolism, though not all enriched amino acid pathways in HMO passed the cut-off for significance but still ranked among the top 25 pathways for that treatment. The metabolic profile of HMO treated Caco2 cells without *S.* Typhimurium 14028 s added supports the gene expression data for HMO treated and infected Caco2 cells, particularly with respect to tryptophan and glutathione metabolism (Figures 4D–F). Though BioMos^®^ treated Caco2 cells also revealed tryptophan metabolites were enriched across 60 min, tryptophan metabolism did not appear as a top expressed genetic pathway in infected cells treated with the same substrate.

### Prebiotic treatment of Caco2 cells drives divergent metabolic gene expression in *Salmonella Typhimurium* 14028

**Figure 5:**
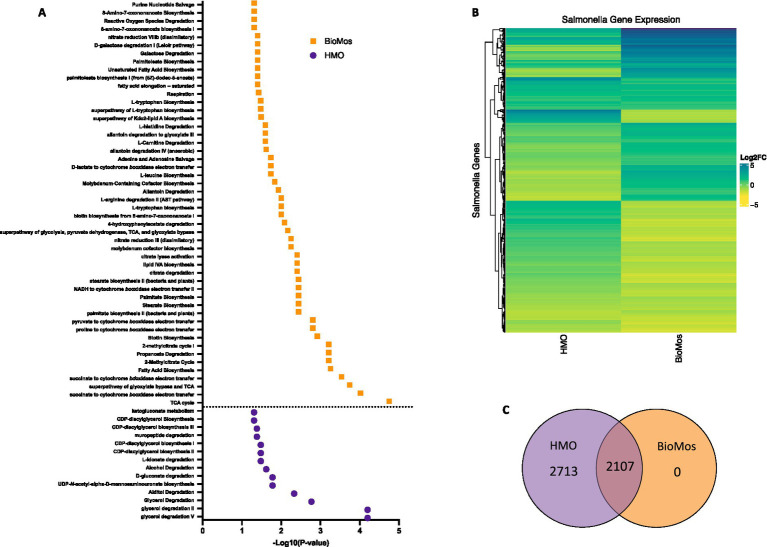
Regulation of gene expression in *S.* Typhimurium 14028 ss in response to addition to Caco2 cells with prebiotic pretreatment. All expression data was collected after 60 min of co-incubation with Caco2 cells, with or without prebiotic treatment and in four biological replicates. **(A)** Enriched metabolic pathway in *S.* Typhimurium added to Caco2 cells pretreated with either BioMos (orange) or HMO (purple). Enrichment determined using BioCyc with a cut of –log10*P* > 1.3 for significance and *S.* Typhimurium added to Caco2 cells and no prebiotic treatment as control condition. **(B)** Broad evaluation of *S.* Typhimurium gene expression in response to either prebiotic condition using log2 fold change data, significance cut off of –log10*P* > 1.3 and Euclidean distance clustering for genes. **(C)** 2,107 genes were significantly expressed in both the HMO and BioMos treated *S.* Typhimurium, while an additional 2,713 unique genes were significantly expressed in the HMO condition and 0 were uniquely expressed in BioMos *S.* Typhimurium.

*Salmonella typhimurium* 14028 s was added to the Caco2-prebiotic mixture 15 min post prebiotic addition and differentially expressed *S.* Typhimurium metabolic pathways were determined for each prebiotic in comparison to *S.* Typhimurium with non-treated Caco2 cells. As seen with the Caco2 cells, significant changes in *S.* Typhimurium gene expression were observed in both HMO and BioMos^®^ treatments, but unlike the host cells, BioMos^®^ treatment did not result in the unique expression of any of the bacterial genes (Figure 5). Both prebiotic treatments significantly (adj. *p* < 0.05) altered the expression of the same 2,107 genes, although HMO altered an additional unique 2,713 genes compared to BioMos^®^. Visualization of all significantly altered genes resulted in a distinct and opposing expression pattern across the two prebiotic treatments, where most genes that are induced in one treatment are repressed in the other (Figure 5). This observation demonstrates the oppositional effect of the prebiotic treatments on *Salmonella*. The stark difference suggests that bacterial metabolism and virulence will be directly related to the type of prebiotic treatment when consumed in the diet that is not predictable and may lead to potentiation of virulence gene expression but repression of the glycan digestion enzymes that effectively reduces association in a healthy epithelial layer. The lack of barrier integrity in inflamed tissue synergizes with the metabolic and virulence modulatory effect of prebiotics on *Salmonella* to promote infection and spread.

Targeted analysis of metabolic gene regulation in *S.* Typhimurium 14028 s identified enrichment of 50 metabolic pathways in the BioMos^®^ treatment and 14 pathways in HMO (Figure 5). HMO and BioMos^®^ treatment did not have any shared enriched pathways in *S.* Typhimurium, revealing distinct reprogramming of metabolism in the pathogen resulting from prebiotic addition. BioMos^®^ treatment resulted in the significant enrichment of multiple amino acid related metabolic pathways including L-tryptophan biosynthesis (−Log_10_ *p* = 1.31), L-leucine biosynthesis (−Log_10_ *p* = 1.74), and L-arginine degradation (−Log_10_ *p* = 2.00). The most significantly enriched pathways in the BioMos^®^ treatment are those related to cellular respiration and energy production. The top four most significant metabolic pathways from the BioMos^®^ treated *S.* Typhimurium were succinate to cytochrome *bd* oxidase electron transfer (−Log_10_ *p* = 3.54), superpathway of glyoxylate bypass and TCA (−Log_10_ *p* = 3.74), succinate to cytochrome *bo* oxidase electron transfer (−Log_10_ *p* = 4.02), and the TCA cycle (−Log_10_ *p* = 4.75). *S.* Typhimurium from the HMO condition did not show enriched expression for any of these metabolic pathways but rather had enriched expression of genes related to CDP-diaglycerol biosynthesis pathways (−Log_10_ *p* > 1.31) and multiple glycerol degradation pathways (−Log_10_ *p* > 1.48). The addition of *S.* Typhimurium to prebiotic pretreated Caco2 cells significantly altered the expression of central metabolic pathways in the pathogen, indicating the addition of these two dietary substrates to host-pathogen systems differentially affects the metabolic communication in host-pathogen metabolic interactions.

### Metabolic profiles reveal distinct shifts in cooperative host-pathogen metabolism after prebiotic treatment

The prebiotic-driven differential expression of genes related to key metabolic pathways in both host and pathogen is supported by the measured metabolites. Whole cell metabolic profiles were determined for each condition (pathogen alone, pathogen and HMO or BioMos^®^, and HMO or BioMos^®^ alone) between two timepoints, 15 min post prebiotic addition and immediately after pathogen inoculation (Time 0) and 60 min post pathogen inoculation (Time 60) with four biological replicates for each combination. K-means clustering with a cluster value of two showed distinct profiles across both time and treatment type (Supplementary Figure 2). Prebiotic type, pathogen inclusion, and sampling time all had marked effects on the metabolic profiles, reflected by the distinct clustering of replicates by treatment combination (Supplementary Figure 3). Caco2 cells with *S.* Typhimurium but without any prebiotic treatment clearly formed two distinct groups. After 15 min of prebiotic incubation (Time 0) there were distinct clusters by prebiotic type, indicating metabolic profiles of the Caco2 cells were rapidly changed by prebiotic incubation irrespective of pathogen presence. As expected, the metabolic profiles of prebiotic treated and infected samples at Time 0 mirrored that of the corresponding T0 profiles of prebiotic treated uninfected Caco2 cells. More detailed comparison of these HMO vs. BioMos^®^ metabolic profiles at Time 0 showed lipids (adj. *p* < 0.02) were increased in the BioMos^®^ treatments as compared to HMO, as were some amino acids like L-kynurenine (adj. *p* = 1.57e-6). The distinct metabolic profiles by prebiotic type observed at Time 0 may be in part due to the addition of exogenous substrates, but combined with the expression data it is clear Caco2 cells alter expression of metabolic pathways in conjunction with prebiotic addition, indicating these noted shifts are not all due to prebiotic addition.

At Time 60 *Salmonella* presence was the dominant driver of metabolic profile shifts, overshadowing the effects of prebiotic pretreatment. HMO and BioMos^®^ treated host cells without *S.* Typhimurium formed their own clusters at Time 60, apart from the samples that included *S.* Typhimurium, while Time 60 samples with *S.* Typhimurium formed a cluster set apart from all others regardless of prebiotic treatment type. Though the metabolic profiles of HMO and BioMos^®^ treated cells with *S.* Typhimurium did not cluster separately on the correlation plot by full profiles, a direct comparison of these two treatments showed notable regulation of iron compounds in the BioMos^®^ condition, including precorrin-4 (adj. *p* = 0.02), cobalt-precorrin-2 (adj. *p* = 0.02), FMNH_2_ (adj. *p* = 0.02), and uroporphyrinogen-III (adj. *p* = 0.02). Though not statistically significant but still notable for respiratory pathways, N_2_-succinylglutamate (adj. *p* = 0.2) and pyrimidine-rings (adj. *p* = 0.2) were both among the top 50 features identified in the comparison of BioMos^®^ plus pathogen to HMO plus pathogen at Time 60.

Across all conditions and time points, 252 metabolites were significant (−log_10_*P* > 1.3) and 64 were not significant using the Kruskal Wallis Test. The significant difference for most metabolites (252/316) across all conditions indicates metabolic fluctuation both over time and by treatment (Supplementary Figure 4). All three treatments (*Salmonella* alone, BioMos^®^ and *Salmonella*, HMO and *Salmonella*) at time 60 formed distinctly different clusters and that were also very different from the prebiotics alone at the same time point. Findings from the correlation plot suggest the presence of *Salmonella* distinctly alters the metabolic profile of the combined host-pathogen metabolome, which supports differing expression patterns of the pathogen in either prebiotic treatment. The additional difference between time 60 metabolomes in the BioMos^®^ and HMO treatments without *Salmonella* affirm the observation that Caco2 cells respond to prebiotic treatment and remodel expression of metabolic pathways irrespective of the presence of microbes.

### Cellular respiration pathways in both host and pathogen are differentially regulated in a prebiotic-dependent manner

**Figure 6:**
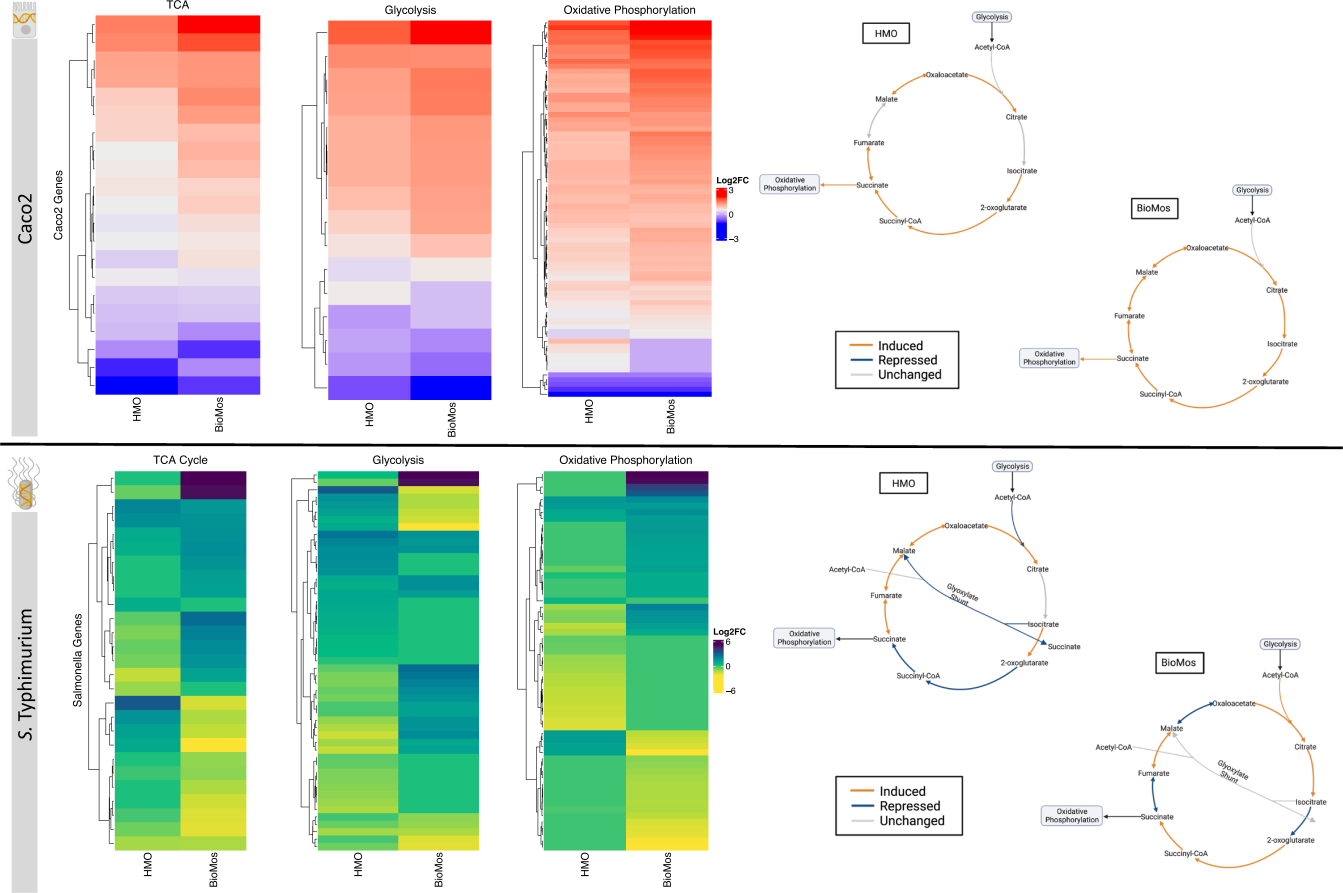
Effect of prebiotic treatment on three major energy-producing metabolic pathways across both infected Caco2 cells and *S.* Typhimurium 14028 ss. Expression of genes related to the TCA cycle, glycolysis and oxidative phosphorylation in response to prebiotic treatment was evaluated for both Caco2 cells (top) and *S.* Typhimurium (bottom). Caco2 expression of these metabolic respiration-related genes was evaluated using *S.* Typhimurium infected Caco2 cells without any prebiotic treatment added as the control for both HMO and BioMos. *S.* Typhimurium expression was determined using *S.* Typhimurium added to Caco2 cells without any prebiotics as control. All heatmaps used Euclidean distance clustering, log2 fold change expression data, and only significant genes with a cutoff of −log10*P* > 1.3. Underlying data for the genetic regulation of the TCA cycle (right) was mapped using IPA for the Caco2 cells and BioCyc for *S.* Typhimurium. All genes represented in orange and blue in the diagrams were significantly expressed compared to the control with a cutoff of –log10*P* > 1.3.

The notable metabolic fluctuations and common theme of differentially regulated energy-metabolism related pathways in both the host and pathogen led to the deeper investigation of three major intertwined metabolic routes: the TCA cycle, glycolysis, and oxidative phosphorylation. Caco2 cells displayed different expression patterns for select genes across these three energy-producing metabolic pathways by prebiotic treatment (Figure 6). However, the effect of prebiotic treatment on the expression of genes related to the TCA cycle, glycolysis, and oxidative phosphorylation was much more pronounced in *S.* Typhimurium (Figure 6).

The TCA cycle in Caco2 cells was similarly expressed in both prebiotic treatments, though BioMos^®^ treated Caco2 cells showed an induction of all major reactions in the cycle while HMO treated cells had no differential expression for the steps from citrate to isocitrate and from fumarate to malate compared to untreated cells. In contrast to Caco2 cells, TCA cycle expression in *S.* Typhimurium is different between the two prebiotic conditions (Figure 6). During infection of HMO treated cells, *S.* Typhimurium displayed an incomplete anaerobic TCA cycle where rather than cycling there is a split into oxidative and reductive branches. The repression of the glyoxylate shunt and the repressed reactions from 2-oxoglutarate to succinate seen in the HMO condition indicate TCA regulation in these *S.* Typhimurium cells is following the pattern of early gut introduction and colonization (Spiga et al., 2017). *S.* Typhimurium infecting BioMos^®^ treated Caco2 cells displayed expression of a more complete TCA cycle, with repression of three major reactions (isocitrate to oxoglutarate, succinate to fumarate, and malate to oxaloacetate) that also serve as entry points for external substrates to continue fueling this cycle. This would result in shifting TCA metabolism toward the production of succinate, which is a metabolite with diverse downstream uses including as an electron acceptor to complete the oxidative steps of the bifurcated *S.* Typhimurium TCA cycle (Spiga et al., 2017; Fan et al., 2022).

Differential regulation of the TCA cycle, and in particular expression of enzymes related to the oxidative TCA cycle seen in the BioMos^®^ treated cells, is supported by the enriched amino acid metabolism observed in *S.* Typhimurium. *S.* Typhimurium in the BioMos^®^ condition showed induced expression of genes related to biosynthesis of the branched chain amino acid leucine (Figure 5). Arginine and histidine degradation were also enriched in BioMos^®^, as was tryptophan biosynthesis. All four amino acids, arginine, histidine, leucine, and tryptophan are precursors for substrates in the TCA cycle and their metabolism contributes to TCA cycle activity, either through entry as pyruvate, acetyl-CoA, or α -ketoglutarate (Spiga et al., 2017). The induction of genes for a complete oxidative TCA cycle in BioMos^®^ treated *S.* Typhimurium in conjunction with the enrichment of amino acid substrates of the TCA cycle suggests complex metabolic regulation by prebiotic treatment and that the regulated pathways do not necessarily confer a host advantage but rather potentiate virulence in the gut environment. Amino acid synthesis in part relies on precursors from another central energy-producing pathway, the pentose phosphate pathway (PPP), so the regulation of this pathway was also examined in more detail.

**Figure 7:**
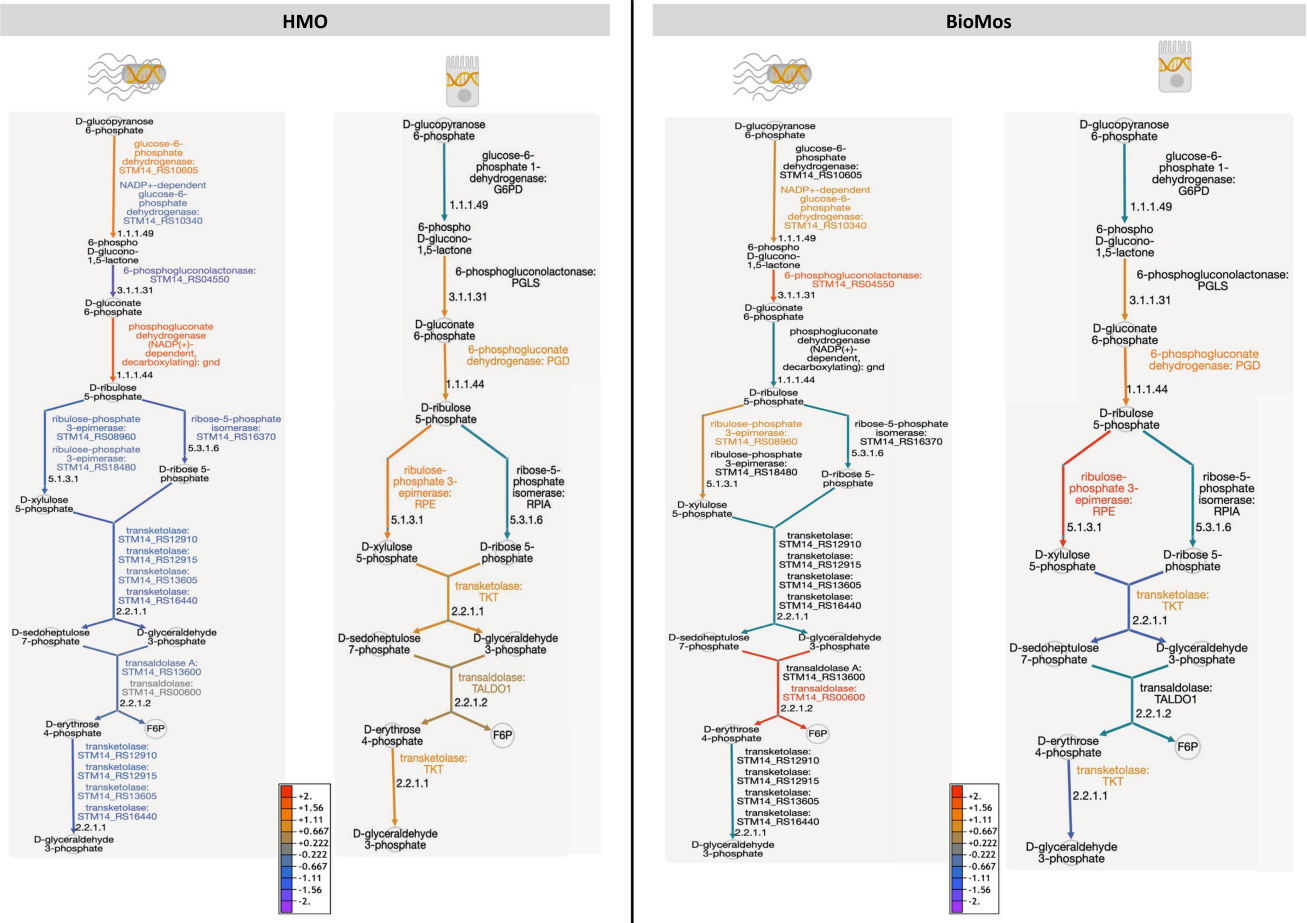
The pentose phosphate pathway is differentially regulated by prebiotic treatment in both Caco2 cells and *S.* Typhimurium 14028 ss. Genetic regulation of the pentose phosphate pathway was evaluated in both S. Tyhpimurium (left within each panel) and Caco2 cells (right within each panel) across HMO addition (left) and BioMos addition (right). Expression is represented as log2 fold change with no prebiotic add matched host/pathogen cells as control and all regulated genes were significant at –log10*P* > 1.3. Increased expression is represented by yellow to red and decreased expression by blue to purple. Black text and teal arrows indicate those genes were non-significant or not annotated in the dataset.

Two other major energy-producing metabolic pathways that run in parallel, glycolysis and the PPP, display this same pattern of pathway regulation as unique to each prebiotic. As seen with the TCA cycle, Caco2 cells had similar patterns of expression across the prebiotic treatments for genes involved in glycolysis, but *S.* Typhimurium expression was distinctly regulated by treatment. Unlike the TCA cycle and glycolysis, regulation of the PPP in the Caco2 cells differed by prebiotic treatment (Figure 7). The PPP is typically induced in host cells during pathogenesis as a means of creating reactive oxygen species (ROS) from NADPH oxidase ultimately decreasing inflammation through the production of anti-inflammatory cytokines (Zhu et al., 2021). Likewise, *Salmonella* utilizes the PPP production of NADPH to control redox balance for survival in the host (Henard et al., 2010). The finding of differential expression of PPP genes by prebiotic treatment coupled to the known importance of PPP regulation in host-pathogen interactions encouraged a deeper evaluation of the expression patterns of this pathway by treatment and cell type.

Detailed mapping of the pathway revealed repression of the reactions involving transketolase in BioMos^®^ treated Caco2 cells, which drives the latter half of the PPP (Figure 7). PPP repression in BioMos^®^ treated Caco2 cells is not seen in *S.* Typhimurium, which instead induced expression of multiple reactions in the PPP, including transaldolase (*STM14_RS00600,* −log_10_*P* = 11.5), which drives production of fructose-6-phopsphate. Contrastingly, HMO treated *S.* Typhimurium displayed repression of the PPP pathway in every step beyond the production of D-ribulose 5-phosphate. Whereas transaldolase was induced in the BioMos^®^ condition, it was repressed in the HMO condition, as was transketolase (*STM14_RS12910*, *STM14_RS12915, STM14_RS16440*) (−log_10_*P* > 2.4). While BioMos^®^ induced multiple parts of the oxidative and non-oxidative branches of the PPP in *S.* Typhimurium, HMO generally repressed the non-oxidative branch of the pathway.

**Figure 8:**
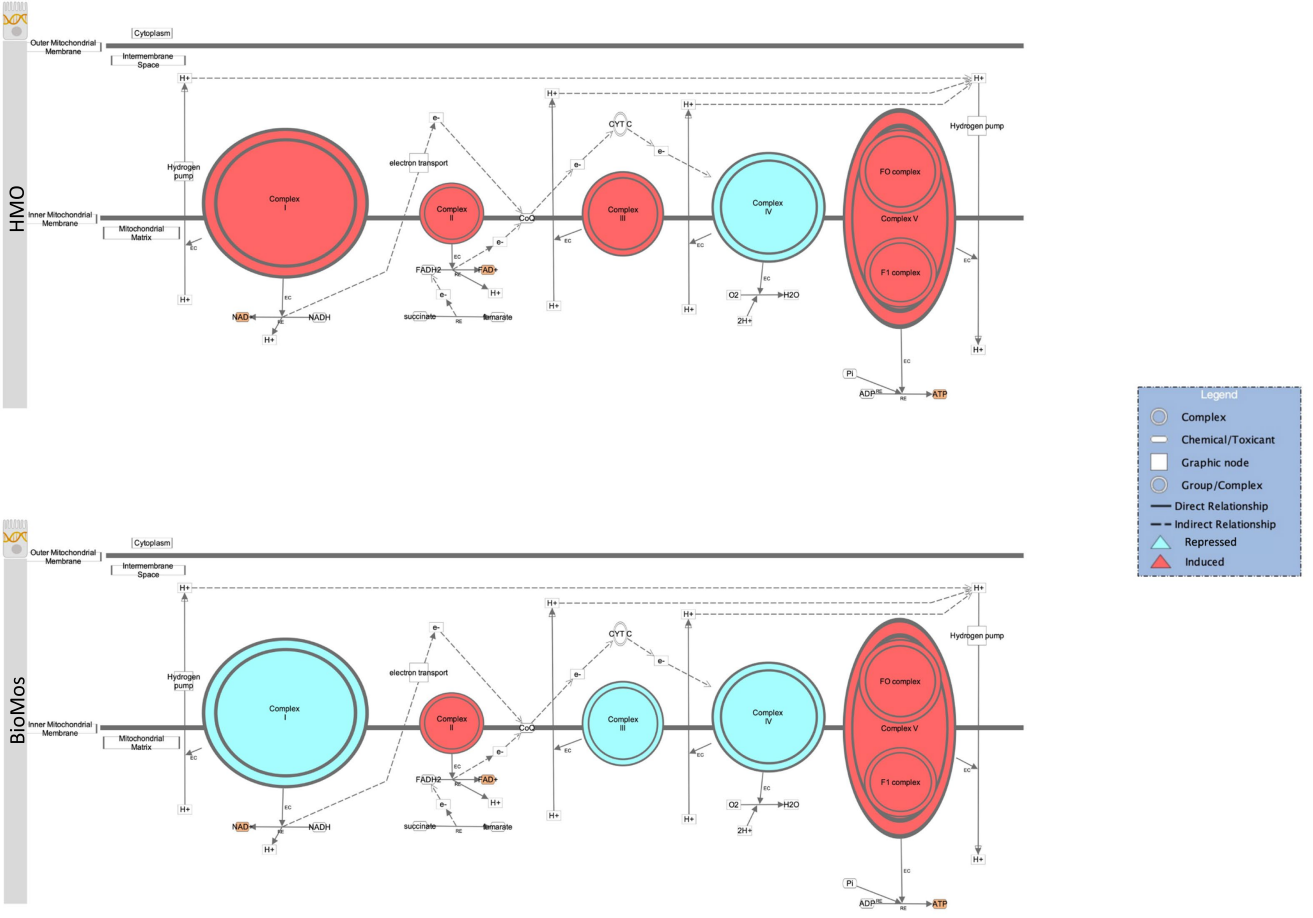
Prebiotic treatment of Caco2 cells challenged with *S.* Typhimurium 14028 ss alters oxidative phosphorylation and expression patterns in the electron transport chain. The canonical oxidative phosphorylation pathway in IPA was overlayed with expression data from Caco2 cells treated with either HMO (top) or BioMos (bottom) and exposed to *S.* Typhimurium for 60 min. Red shading in the complexes indicates induction of genes responsible for that complex’s activity and teal shading indicates repression of that complex. Orange shading on the products indicates a predicted increase in concentration due to the expression pattern.

The final energy producing pathway investigated in this study likewise followed the same trajectory as the two prior paths. Oxidative phosphorylation in both the pathogen and in the Caco2 cells showed distinct modulation of gene expression related to prebiotic treatment. A comprehensive examination of gene expression related to oxidative phosphorylation in Caco2 cells revealed a subset of genes related to complex I of the mitochondrial respiratory chain (*NDUFA3, NDUFA7, NDUFB10, NDUFS6,* and *NDUFS8*) that were repressed in the BioMos^®^ condition (Figure 8). Further, mapping of the respiratory chain gene expression showed repression of Complex IV in both HMO and BioMos^®^ treated cells, but additional repression of Complex I in BioMos^®^. Notably both prebiotic conditions altered the expression of multiple electron shuttling pathways including the electron transport chain directly.

## Methods

A graphical representation of the experimental set up can be found in Supplementary Figure 5.

### Oligosaccharides

HMO was isolated and given as a gift to the Weimer lab by Dr. Daniela Barile (UC Davis, CA, United States) (Strum et al., 2012). BioMos^®^ is a yeast-derived and commercially available product from Alltech Inc. (Nicholasville, KY, United States). Brief descriptions of each oligosaccharide mixture can be found in Table 3. Both oligosaccharides were made into a 1% working concentration in high glucose DMEM (HyClone Laboratories, Logan, UT, United States).

**Table 3:** Source and content of oligosaccharides used in this experiment.

Prebiotic	Main oligosaccharide structure(s)	Prebiotic source
BioMos^®^	Mannan-oligosaccharides	Proprietary *Saccharomyces cerevisiae* derived product
HMO	Five monosaccharides (Glucose, Galactose, N-acetylglucosamine, Fucose, Sialic Acid) combined into complex oligosaccharides	Extraction from human breast milk

### Bacterial strain and growth conditions

Bacterial cells were grown as previously described (Arabyan et al., 2016; Park et al., 2016; Arabyan et al., 2017; Arabyan et al., 2017; Heithoff et al., 2012). Briefly, *Salmonella enterica* subsp. *enterica* serovar Typhimurium strain LT2 was grown and used for the initial prebiotic adhesion and invasion as well as the glycosyl hydrolase knockout experiments due to its lessend virulence compared to *Salmonella enterica* subsp. *enterica* serovar Typhimurium strain 14028 s. *S.* Typhimurium strain 14028 s was used for an follw-up adhesion and invasion assays and related gene expression experiments due to its increased virulence phenotype compared to *S.* Typhimurium strain LT2 and its ability to better mimic virulent *in vivo Salmonella* infections. All *Salmonella* were grown in LB (Difco, BD, Thermo Scientific, Rockford, IL, United States) at 37 °C, shaking at 220 rpm for 14–16 h prior to each use. Glycosyl hydrolase enzyme knockouts of *malS* and *nanH* were made as described previously (Arabyan et al., 2016)

### Human cell line and growth conditions

Human colonic carcinoma (Caco2) cell lines were obtained from ATCC (HTB-37) and grown as described previously (Chen et al., 2017). Briefly, Caco2 cells were thawed from liquid nitrogen stocks stored in DMEM with 10% DMSO then grown in DMEM with 10 mM MOPS (Sigma, St. Louis, MO, United States), 10 mM TES (Sigma, St. Louis, MO, United States), 15 mM HEPES (Sigma, St. Louis, MO, United States), 2 mM NaH_2_PO_4_ (Sigma, St. Louis, MO, United States), 20% fetal bovine serum (HyClone Laboratories), 1% glutamax (Thermo Scientific, Rockford, IL, United States), 1% PenStrep (Thermo Scientific, Rockford, IL, United States) and 1% non-essential amino acids (Thermo Scientific, Rockford, IL, United States). Culture medium was renewed every 3 days. Caco2 cells were seeded at 10,000 cells/cm^2^ into 96-well plates then differentiated for 12–15 days for gentamicin protection assays.

### *In vitro* colonic cell infection assays

Colonic cell infection assays were also performed according to Chen et al. (2017) and Arabyan et al. (2016), which adapted methods from Shah et al. (2013) and Shah et al. (2014). Oligosaccharides suspended at 1% (w/v) in serum-free DMEM were added to differentiated Caco2 cells and incubated for 15 min. Following prebiotic pretreatment, stationary phase *S.* Typhimurium (*n* = 3 biological replicates; multiplicity of infection = 1,000) was added to the pretreated Caco2 cells and incubated for 60 min. PBS buffer (pH = 7.2) was used to wash the cells and 50 mL Warnex buffer (AES Chemunex Canada, Inca, Montrealm QC, Canada) was applied to lyse the cells according to the manufacturer’s directions. Deactivation of Warner lysis was done with a 15 min incubation at 95°C. Samples were then diluted 1:10 in nuclease-free water and stored at −20°C for qPCR quantification. Quantification of *S.* Typhimurium LT2 and 14028 sS cells was done via qPCR using primers previously validated by Arabyan et al. (2016), F: 5′- ACG CGG 313 TAT CAT CAA AGT GG - 3′; R: 5′ - ATC GGG TGG ATC AGG GTA AC - 3′. Significant differences in association across control and treatment were estimated using one-way ANOVA with Tukey test and graphed in GraphPad Prism V9 (GraphPad Software Inc., La Jolla, CA, United States).

### Metabolomics

Two different analytical setups for liquid chromatography coupled to mass spectrometry (LCMS) were used to survey a wider variety of non-volatile compounds. Samples were split between two capped LC vials, then were stored at −20°C prior to analysis. Non-volatile compounds from the culture supernatant were analyzed via LC–MS using a hydrophilic interaction chromatography (HILIC) column for hydrophilic and polar molecules and a reversed-phase (RP) column for nonpolar molecules according to the method by Borras et al. (2017) and Aksenov et al. (2017). All samples were analyzed both via HILIC and RP on an Agilent 1,290 series ultrahigh-performance LC system with an Agilent 6,230 time-of-flight (TOF) mass spectrometer (Agilent Technologies, Santa Clara, CA, United States). An Agilent Jet Stream (AJS) nebulizer was used working in positive mode (+) and acquiring a mass range between 50 and 1,700 Thomson (m/z) at 4 spectra/s and high-resolution mode. Sheath gas temperature was 350°C, gas flow was 11 L/min, and fragmentor voltage was set at 120 V. A model 6,545 quadrupole TOF mass spectrometer (Agilent Technologies, Santa Clara, CA, United States) was used for final MS/MS compound identification. Water, acetonitrile, and 10% acetonitrile suspension mix were analyzed as blanks alongside the samples, which were all analyzed via injection of 5 μL aliquots with samples housed in an autosampler maintained at 4 °C. All LCMS analysis was performed with four biological replicates.

HILIC samples were analyzed on an Acuity UPLC bridged ethyl hybrid amide column (130 Å, 1.7 μm, 2.1 mm × 100 mm; Water, Milford, MA, United States). Water (A) and 90% acetonitrile in water (B) were used as mobile phases, both at pH 5 with ammonium acetate and acetic acid buffer. A linear gradient from 0 to 10% A was applied post-injection in 20 min with a flow rate of 0.3 mL/min. The flow rate was then reduced to 0.2 mL/min and phase A was increased to 95% in 10 min, for a total analysis time of 41 min. HILIC quality controls were a Water 1,806,006,963 HILIC QC (Waters, Milford, MA, United States) and a custom-made QC. The custom QC consisted of 5 μM carnitine, lysine, adenylputricine, aminocaproic acid, ornithine, tigonelline, alaninol, acetylcarnitine, 1-(2-pyramidyl) piperazine, methoxychalcone, cholecalciferol, 13-docosenamide and oleamide.

RP samples were analyzed on a Poroshell 120 EC-C18 column (2.7 μm, 3.0 mm × 50 mm; Agilent Technologies, Wilmington, DE, United States) at 30 °C. A mix of 1% phase A (60% acetonitrile in water) and 99% phase B (10% acetonitrile in isopropanol), both containing 10 mM of ammonium formate and formic acid, was used as the initial mobile phase. The total analysis time was 24 min with a flow rate at 0.3 mL/min, which consisted of phase B gradient reaching 30% post injection in 4 min, then rising to 48% B in 1 min, 82% B in 17 min, and 99% B in 1 min. The quality control was a standard solution Waters 6,963 RP QC (Waters, Milford, MA, United States) and was injected along with the samples.

Agilent Mass Hunter Qualitative Analysis B.05.00SP1 software was used to examine the total ion LCMS chromatograms. The “Find by Molecular Feature” algorithm was used within a mass range from 50 to 1,700 Da for peak deconvolution. Molecular feature abundance was evaluated through integration of the extracted compound chromatograms (ECC) of the corresponding ions and then exported to .cef format. Mass Profiler Professional 12.1 software was used for peak alignment with a mass window of 40 ppm, 26mDa, and a retention time shift of 0.5 and 1 min for HILIC and RP, respectively. A peak table was made containing retention time in mins, molecular mass, and the intensity values (peak area) for each sample.

Resulting metabolic data was analyzed using MetaboAnalyst 5.0 (Pang et al., 2022). Comparisons between treatments and across timepoints were made using both the statistical analysis [one factor] function and enrichment analysis modules. Samples were normalized by median, then all data was log_10_ transformed and scaled by mean-centering and standard deviation. Metabolite set enrichment analysis (MSEA) was performed using the KEGG database for reference.

### RNA extraction

The 100 K Pathogen Genome Project bacterial protocol (Kong et al., 2013) was used to extract *S.* Typhimurium RNA from the infection assays, while host cells were lysed by passaging cells through a 22-gauge needle (Guo et al., 2010). Combined host and pathogen cells were pelted via centrifugation then suspended in Trizol LS Reagent (Cat #10296028, Thermo Fisher Scientific, Waltham, MA, United States). Host and pathogen RNA was extracted from the Trizol LS suspension following manufacturer’s instructions. The BioAnalyzer RNA kit (Agilent Technologies Inc., Santa Clara, CA, United States) and Nanodrop (Nanodrop Technologies, Wilmington, DE, United States) were used to confirm RNA purity (A_260/230_ and A_260/280_ ratios ≥1.8, ≤2.0) and integrity.

### RNAseq library preparation

RNAseq library preparation was performed exactly as outline in Chen et al. (2017). Briefly, the SuperScript Double-Stranded cDNA Synthesis kit (11917-010; Invitrogen, Carlsbad, CA, United States) was used to synthesize double-stranded cDNA following the manufacturer’s instructions. A NanoDrop 2,000 spectrophotometer (Nanodrop Technologies, Wilmington, DE, United States) an Agilent 2,100 BioAnalyzer (Agilent Technologies Inc., Santa Clara, CA, United States) was used to assay cDNA quality. All sequencing was done with four biological replicates in each condition.

The Kapa HyperPlus library preparation kit (kk814, KAPA Biosystems, Boston, MA, United States) with BIOO Scientific NEXTFlex adaptors (514,105, BIOO, Austin, TX, United States) were used in the construction of the sequencing library. Library concentration was measured using the KAPA SYBR FAST qPCR kit Master Mix (2x) Universal (kk4903; KAPA Biosystems, Boston, MA, United States) on Bio-Rad CFX96 (Bio-Rad Laboratories, Hercules, CA, United States) and fragment size distribution was assessed using the High Sensitivity kit (Agilent Technologies Inc., Santa Clara, CA, United States). Libraries were indexed at eight libraries per lane and sequenced with PE150 on a HighSeq4000 at the California Institute for Quantitative Biosciences in the Vincent J Coates Genomics Sequencing Lab (Berkeley, CA, United States).

### Statistical analysis for differential gene expression

Sequence processing and analysis was done following the methods in Chen et al. (2017). Sequence files can be found on NCBI SRA under BioProject PRJNA1302535. Raw sequence reads were first trimmed with Trimmomatic (Bolger et al., 2014) then aligned to the Ensembl GRCh38 human genome using HISAT2 (Kim et al., 2019) with an index downloaded on 07/16/22 (grch38_tran). Alignment was done in paired-end mode with soft clippings permitted and paired-end reads that did not map to the human genome were separated. Reads that did not align to the human genome were subsequently aligned using Bowtie2 (Langmead and Salzberg, 2012) to the *Salmonella enterica* genome (GCA_003253385.1_ASM325228v1). Samtools (Li et al., 2009) was used to compress alignment files from HISAT2 and Bowtie2 for output to differential expression analysis.

Differential expression analysis was performed in edgeR (Robinson et al., 2010) from gene counts estimated by featureCounts in the Rsubread R package (Liao et al., 2014). Human gene counts were produced using the Ensembl GRCh38.86.gtf annotation and *S.* Typhimurium 14028 s counts were generated from GCA_003253385.1_ASM325228v1.gtf annotation file. Human and *S.* Typhimurium gene count tables were entered separately into edgeR for normalization and differential expression. Genes with counts per million less than one and with expression in fewer than two samples per group were discarded and reads were normalized using the library size via the normLibSizes() function in edgeR. Treatment groups contained pairwise comparisons, so the edgeR exact test was used for differential expression estimation. Significance was defined as adjusted *p*-value (FDR, this was done using a Bonferroni correction) of less than or equal to 0.05 or −log10 *p*-value greater than or equal to 1.3. No reads aligned to *Salmonella* from uninfected cells so no differential expression analysis was done for *Salmonella* reads in uninfected cells.

### Expression data for Caco2 cells

Qiagen’s Ingenuity Pathway Analysis software version 01-22-01 (IPA, Qiagen, Redwood City, CA, United States) was used to determine canonical pathways differentially expressed in Caco2 cells by prebiotic treatment. Canonical pathway mapping was performed in IPA and overlayed with experimental data. Expression data for TLRs was plotted using Prism 9 (GraphPad Software, La Jolla, CA, United States). Heatmaps were made using R Version 4.2.2 “Innocent and Trusting” along with the ComplexHeatmap Package version 2.16.0 (Gu et al., 2016). All other plots related to expression data not from IPA were made using Prism 9 (GraphPad Software, La Jolla, CA, United States).

### Expression data for *S*. Typhimurium

*S.* Typhimurium differential expression data was uploaded to BioCyc SmartTables for further analysis (Pathway Tools version 27, SRI International, Menlo Park, CA, United States) (Karp et al., 2019). Determination of enriched pathways was done using a two-tailed Fisher’s exact test (*p* ≤ 0.05) with the Pathway Tools *S.* Typhimurium strain 14028 s genome. Enriched pathways from were plotted using Prism 9 (GraphPad Software, La Jolla, CA, United States) and heatmaps were made using R Version 4.2.2 “Innocent and Trusting” along with the ComplexHeatmap Package version 2.16.0 (Gu et al., 2016).

## Discussion

Targeted application of probiotics *in vitro* altered gene expression and metabolism of both host and pathogen (Caco2 and *S.* Typhimurium, respectively) in a prebiotic-specific manner. This observation amends the current paradigm that prebiotics exert protective effects through catabolism by commensal gut microbiota (Oba et al., 2020) and mitigate infection through competitive exclusion (Kerr et al., 2013). Though both prebiotics in this study were able to reduce host-association at 1% addition, the path by which HMO and BioMos^®^ drove expression changes in host Caco2 and pathogen cells were prebiotic-specific. Intriguingly, both prebiotics induced the expression of different virulence-modulating two-component systems (TCS) and other well-documented virulence factors like SPI-1 and SPI-2. Previous work from our lab illustrated the same prebiotics used here remodeled the epithelial cell surface and modulated the expression of receptors, priming the colonic epithelial cells for response to infection with *L. monocytogenes* (Chen et al., 2017; Arabyan et al., 2016; Marcobal et al., 2011; Park et al., 2016; Maga et al., 2013). Together these results indicate that rather than acting as an inert physical blockade, host-protective effects of the dietary prebiotics tested in this study stemmed from a complex orchestration of differential expression of bacterial virulence factors, host membrane receptors, and shared metabolism.

In line with this prebiotic-specific regulation, one striking observation in the HMO condition was the repression of membrane receptor CpxA alongside induction of the ArcA regulator. Because the CpxAR and ArcBA systems cross-regulate, with CpxA able to phosphorylate ArcA (Cudic et al., 2017), this shift may have transiently pushed *S.* Typhimurium toward cell death early in infection, though subsequent repression of CpxA suggests this pathway was later attenuated (Shaw et al., 2024). At the same time, although multiple virulence factors including SPI-1 were induced, reduced expression of glycosyl hydrolases limited the bacterium’s ability to degrade the protective mucosal glycoprotein barrier and gain access to epithelial receptors. In a healthy gut, this mucus layer provides a critical first line of defense against enteric pathogens (McGuckin et al., 2011), but *S.* Typhimurium typically circumvents this barrier by producing glycosyl hydrolases that cleave glycoproteins and oligosaccharides, thereby exposing the host cell surface (Arabyan et al., 2016). The suppression of these enzymes in the HMO treatment suggests that even with virulence factor induction, *S.* Typhimurium was not able to execute the essential first step of breaching the mucosal barrier. These findings align with our previous observations that HMO remodels epithelial surfaces and modulates receptor availability (Chen et al., 2017; Arabyan et al., 2016; Marcobal et al., 2011; Park et al., 2016; Maga et al., 2013), suggesting that HMO disrupts both bacterial regulation and host accessibility. Thus, while HMO exposure triggered significant shifts in TCS signaling and virulence factor induction, the combined pathogen and host responses prevented *S.* Typhimurium from achieving successful invasion under these conditions.

Host modulation of surface receptors driven by prebiotic treatment similarly contributed to the regulation of host-pathogen association. Prebiotic treatment led to the repression of multiple epithelial-associated TLRs (Abreu et al., 2005), with only one receptor, TLR2, induced. Previous work noted that increased TLR2 expression exacerbates *S.* Typhimurium infection through negative regulation of nitric oxide synthase expression and a reduction in epithelial barrier integrity (Zhan et al., 2015). Expression of TLR-related signaling molecule MYD88 was also induced in both prebiotic conditions. Previous studies using mesenchymal stems cells, in which *Salmonella* can intracellularly persist and transit through the body (Mohamad-Fauzi et al., 2023), found *MYD88* expression was induced in these tri-lineage cells (Kim et al., 2017). The induction of *MYD88* in mesenchymal stem cell by the Type III Secretion System (T3SS) resulted in the systemic spread of the pathogen through the host (Kim et al., 2017), illustrating the exploitation of a host immune response for pathogenic gain and is notable given this same signaling molecule was induced here for Caco2 cells in both prebiotic conditions.

HMO and BioMos^®^ reduced *S.* Typhimurium adherence to colonic epithelial cells, but this effect was not fully explained by virulence factor repression or receptor blocking. Modulation of energetic pathways in both host and pathogen offers one possible mechanism. While metabolic composition differed across treatments and timepoints, BioMos^®^ consistently drove distinct clustering of host and pathogen metabolites, with uninfected Caco2 cells at T60 separating clearly from all other samples. However, infection overrode this BioMos^®^-driven profile, suggesting its protective effects may be transient and dependent on infectious dose and gut condition. BioMos^®^ is widely used in livestock to promote health (Spring et al., 2015), but the reduced efficacy observed here in a human gut model indicates species-specific differences that warrant further study.

The observed metabolic divergence between treatments is further supported by transcriptomic data, which indicate that presence of BioMos^®^ in the media drives metabolic shifts not observed in the HMO condition. The forward push of the TCA cycle reactions, along with the repression of the PPP, and induction of ubiquinone-related metabolism to fuel the mitochondrial electron transport chain in BioMos^®^ treated *S.* Typhimurium suggests BioMos^®^ encouraged aerobic respiration (Unden et al., 2014) and improved energy production for the pathogen. The TCA cycle in BioMos^®^ treated *S.* Typhimurium displayed a push toward the production of succinate and repression of the downstream production of fumarate, an intriguing finding since succinate is a central compound in driving pathogenesis of multiple enteric organisms (Spiga et al., 2017; Ferreyra et al., 2014). Previous work in *Clostridium difficile* coupled with commensal *Bacteroides thetaiotaomicron* revealed the increased production of succinate by *B. thetaiotaomicron* after antibiotic disturbance supported the proliferation and pathogenesis of *C. difficile* in mice (Ferreyra et al., 2014). In this *C. difficile* study, succinate was found to be utilized as a substrate for the regeneration of NAD + (Ferreyra et al., 2014) and succinate has likewise been shown to support increased gut colonization and pathogenesis by *S.* Typhimurium (Spiga et al., 2017). The control of redox metabolism in conjunction with amino acid metabolism in the gut by *S.* Typhimurium is a key driver of pathogenesis and the switch to aerobic respiration pathways in the BioMos^®^ condition suggests virulence-favoring conditions for *S.* Typhimurium (Lee et al., 2022).

At the same time, while BioMos^®^ treatment moderated host cell metabolism related to immunological responses, such as reduced calcium transport and expression of multiple lipid-related pathways (Yeung et al., 2019), the addition of *Salmonella* appeared to overwhelm the host protective responses. This takeover may be explained by the prebiotic composition. As BioMos^®^ is a commercial product derived from the cell walls of *Saccharomyces cerevisiae*, it is not pure lab-extracted MOS but instead contains additional substrates derived from the yeast including metal ions and amino acids (Spring et al., 2015). *S.* Typhimurium expression showed significant enrichment of amino acid related pathways, which may be a result of these other substrates and not MOS itself. This idea of off target effects from mixed substrates is bolstered by the more limited metabolic findings in the HMO treatment. As HMO is not commercially available, the substrate used in this experiment was extracted in a lab was a pure mixture of complex oligosaccharides. The dichotomy of commercial MOS and pure HMO in the experiment, along with the subsequent observation of differing effects, highlight the off-target effects of commercial prebiotic products may depend on oligosaccharide product composition and requires further research to gauge the range of possible effects.

Amino acid regulation is a key component of nutritional immunity between hosts and pathogens (Ren et al., 2018). Amino acid-derived compounds, such as fumarate, can act as alternate electron acceptors and contribute to energetically favorable aerobic respiration (Nguyen et al., 2020; Margolis et al., 2023), suggesting the regulation of amino acid metabolism in the BioMos^®^ condition could support pathogenic colonization. Arginine metabolism was enriched in *Salmonella* in the BioMos^®^ condition. Arginine serves as a key substrate for proline production and proline is known to modulate oxidative stress in *Salmonella* over the course of infection (Christgen and Becker, 2019). L-tryptophan, an essential amino acid and central substrate for many bioactive molecules and a key precursor for nicotinamide adenine dinucleotide (NAD^+^), was also regulated in both host and pathogen by BioMos^®^ treatment (Covarrubias et al., 2021; Hu et al., 2022; Bosi et al., 2020). L-tryptophan biosynthesis was upregulated in BioMos^®^ treated *Salmonella* and generalized tryptophan metabolism was an enriched metabolic pathway in Caco2 cells. L-kynurenine, a direct production of tryptophan degradation, was an enriched metabolite in BioMos^®^ treatment as compared to HMO. L-kynurenine is a neuroactive metabolite with broad protective and detrimental neurological effects in humans (Shaw et al., 2023). This additional finding of tryptophan regulation by both host and pathogen in conjunction with the more specific measurements of metabolites from this metabolic pathway gives credence to prebiotics potentiating health effects through regulation of host metabolism (Bedu-Ferrari et al., 2022). Though intriguing, more research is needed on the potential systemic effects of metabolic modulation from prebioitic administration.

HMO treatment better prevented *S.* Typhimurium association with host epithelial cells compared to BioMos^®^ and primarily resulted in anaerobic respiration in the pathogen. Somewhat confoundingly expression data for *S.* Typhimurium showed more uniquely expressed genes in HMO (2713) and no uniquely expressed genes in BioMos^®^. The complexity of the HMO oligosaccharides in contrast with the simpler MOS structure and accompanying yeast cell wall components may provide an explanation for this finding. Indeed, HMO treatment did not result in the enrichment of many metabolic pathways for *Salmonella* and alterations in host cell expression were minimal. The repression of energy production pathways in *Salmonella* with HMO treatment, known host cell surface remodeling by HMO (Chen et al., 2017), along with previously understood positive immunomodulatory functions of this prebiotic (Rousseaux et al., 2021) supports that HMO in healthy gut condition may attenuate enteric infection by *Salmonella.* Pathogenic attenuation by dietary substrates appears highly substrate and host specific, as illustrated by one clinical trial applying FOS to patients with Crohn’s Disease, which produced no clinical benefit to participants (Benjamin et al., 2011). This previous work provides some *in vivo* evidence that prebiotics in an inflamed gut may not always produce the expected ameliorative properties.

Observations from this work indicate dietary substrates may on the surface attenuate virulence but underneath can drive expression of pathogenic-related genes and metabolic pathways; a cautionary finding for the clinical application of dietary interventions. The observations of altered virulence and metabolic expression in this experiment are intriguing, but pathogenic effects will require further validation using more complex *in vitro* models such as organoids (Shaw et al., 2025) or animal models (Azad et al., 2020). The focused cell culture model used in this work is beneficial for uncovering mechanistic interactions between dietary substrates, microbes, and host cells, however the model is limited in its ability to uncover more complex holistic host responses, such as those involving the immune system.

The results from the focused model used here indicate the beneficial effects of dietary prebiotics may be contingent on their addition to an already healthy gut environment, and rescue of a dysbiotic or inflamed gut from infection is specific to only select oligosaccharides. In the context of pathogens, the modulation of invasion by the prebiotic oligosaccharides seen in this study shows promise, but the diverging effects on pathogenic energy metabolism supports the ongoing need for substrate-specific studies across both healthy and dysbiotic gut environments.

## Data Availability

The datasets presented in this study can be found in online repositories. The names of the repository/repositories and accession number(s) can be found at: https://www.ncbi.nlm.nih.gov/bioproject/PRJNA1302535.

## References

[ref1] Abreu M. T. Fukata M. Arditi M. (2005). TLR signaling in the gut in health and disease. J. Immunol. 174, 4453–4460. doi: 10.4049/jimmunol.174.8.4453 15814663

[ref2] Aksenov A. A. Zamuruyev K. O. Pasamontes A. Brown J. F. Schivo M. Foutouhi S.. (2017). Analytical methodologies for broad metabolite coverage of exhaled breath condensate. J. Chromatogr. B Analyt. Technol. Biomed. Life Sci. 1061-1062, 17–25. doi: 10.1016/j.jchromb.2017.06.038, PMID: 28697414 PMC5573623

[ref3] Arabyan N. Huang B. C. Weimer B. C. (2017). Amylases and their importance during glycan degradation: genome sequence release of Salmonella amylase knockout strains. Genome Announc. 5:e00341-17. doi: 10.1128/genomeA.00355-17, PMID: 28522713 PMC5477324

[ref4] Arabyan N. Weis A. M. Huang B. C. Weimer B. C. (2017). Implication of Sialidases in Salmonella infection: genome release of Sialidase knockout strains from *Salmonella enterica* Serovar Typhimurium LT2. Genome Announc. 5. doi: 10.1128/genomeA.00341-17, PMID: 28495784 PMC5427219

[ref5] Arabyan N.. (2016). Salmonella degrades the host Glycocalyx leading to altered infection and glycan remodeling. Sci. Rep. 6:29525. doi: 10.1038/srep29525 27389966 PMC4937416

[ref6] Azad M. A. K. Gao J. Ma J. Li T. Tan B. Huang X.. (2020). Opportunities of prebiotics for the intestinal health of monogastric animals. Anim Nutr 6, 379–388. doi: 10.1016/j.aninu.2020.08.001, PMID: 33364453 PMC7750794

[ref7] Bedu-Ferrari C. Biscarrat P. Langella P. Cherbuy C. (2022). Prebiotics and the human gut microbiota: from breakdown mechanisms to the impact on metabolic health. Nutrients 14:2096. doi: 10.3390/nu14102096 35631237 PMC9147914

[ref8] Benjamin J. L. Hedin C. R. H. Koutsoumpas A. Ng S. C. McCarthy N. E. Hart A. L.. (2011). Randomised, double-blind, placebo-controlled trial of fructo-oligosaccharides in active Crohn's disease. Gut 60, 923–929. doi: 10.1136/gut.2010.232025, PMID: 21262918

[ref9] Bode L. (2012). Human milk oligosaccharides: every baby needs a sugar mama. Glycobiology 22, 1147–1162. doi: 10.1093/glycob/cws074 22513036 PMC3406618

[ref10] Bolger A. M. Lohse M. Usadel B. (2014). Trimmomatic: a flexible trimmer for Illumina sequence data. Bioinformatics 30, 2114–2120. doi: 10.1093/bioinformatics/btu170, PMID: 24695404 PMC4103590

[ref11] Borras E. Aksenov A. A. Baird M. Novick B. Schivo M. Zamuruyev K. O.. (2017). Exhaled breath condensate methods adapted from human studies using longitudinal metabolomics for predicting early health alterations in dolphins. Anal. Bioanal. Chem. 409, 6523–6536. doi: 10.1007/s00216-017-0581-6, PMID: 29063162

[ref12] Bosi A. Banfi D. Bistoletti M. Giaroni C. Baj A. (2020). Tryptophan metabolites along the microbiota-gut-brain Axis: an Interkingdom communication system influencing the gut in health and disease. Int J Tryptophan Res 13:1178646920928984. doi: 10.1177/1178646920928984, PMID: 32577079 PMC7290275

[ref13] Catron D. M. Lange Y. Borensztajn J. Sylvester M. D. Jones B. D. Haldar K. (2004). *Salmonella enterica* serovar Typhimurium requires nonsterol precursors of the cholesterol biosynthetic pathway for intracellular proliferation. Infect. Immun. 72, 1036–1042. doi: 10.1128/IAI.72.2.1036-1042.2004, PMID: 14742551 PMC321618

[ref14] Chen P.. (2017). Prebiotic oligosaccharides potentiate host protective responses against *L. monocytogenes* Infection. Pathogens 6:68. doi: 10.3390/pathogens6040068, PMID: 29257110 PMC5750592

[ref15] Cheng L. Reiter T. Huang B. Kong N. Weimer B. C. (2019). Human milk oligosaccharides and its acid hydrolysate LNT2 show immunomodulatory effects via TLRs in a dose and structure-dependent way. J. Funct. Foods 59, 174–184. doi: 10.1016/j.jff.2019.05.023

[ref16] Cheon S. Kim G. Bae J. H. Lee D. H. Seong H. Kim D. H.. (2023). Comparative analysis of prebiotic effects of four oligosaccharides using in vitro gut model: digestibility, microbiome, and metabolome changes. FEMS Microbiol. Ecol. 99:fiad002. doi: 10.1093/femsec/fiad002, PMID: 36623850 PMC9875365

[ref17] Christgen S. L. Becker D. F. (2019). Role of Proline in pathogen and host interactions. Antioxid. Redox Signal. 30, 683–709. doi: 10.1089/ars.2017.7335, PMID: 29241353 PMC6338583

[ref18] Commane D. M. Shortt C. T. Silvi S. Cresci A. Hughes R. M. Rowland I. R. (2005). Effects of fermentation products of pro- and prebiotics on trans-epithelial electrical resistance in an in vitro model of the colon. Nutr. Cancer 51, 102–109. doi: 10.1207/s15327914nc5101_14, PMID: 15749636

[ref19] Covarrubias A. J. Perrone R. Grozio A. Verdin E. (2021). NAD(+) metabolism and its roles in cellular processes during ageing. Nat. Rev. Mol. Cell Biol. 22, 119–141. doi: 10.1038/s41580-020-00313-x, PMID: 33353981 PMC7963035

[ref20] Craft K. M. Townsend S. D. (2018). The human Milk Glycome as a defense against infectious diseases: rationale, challenges, and opportunities. ACS Infect Dis 4, 77–83. doi: 10.1021/acsinfecdis.7b00209, PMID: 29140081 PMC6011826

[ref21] Cudic E. Surmann K. Panasia G. Hammer E. Hunke S. (2017). The role of the two-component systems Cpx and arc in protein alterations upon gentamicin treatment in *Escherichia coli*. BMC Microbiol. 17:197. doi: 10.1186/s12866-017-1100-9, PMID: 28923010 PMC5604497

[ref22] Dillon S. Agrawal S. Banerjee K. Letterio J. Denning T. L.. (2006). Yeast zymosan, a stimulus for TLR2 and dectin-1, induces regulatory antigen-presenting cells and immunological tolerance. J. Clin. Invest. 116, 916–928. doi: 10.1172/JCI27203, PMID: 16543948 PMC1401484

[ref23] Fan H. H. Fang S. B. Chang Y. C. Huang S. T. Huang C. H. Chang P. R.. (2022). Effects of colonization-associated gene yqiC on global transcriptome, cellular respiration, and oxidative stress in *Salmonella Typhimurium*. J. Biomed. Sci. 29:102. doi: 10.1186/s12929-022-00885-0, PMID: 36457101 PMC9714038

[ref24] Ferreyra J. A. Wu K. J. Hryckowian A. J. Bouley D. M. Weimer B. C. Sonnenburg J. L. (2014). Gut microbiota-produced succinate promotes *C. difficile* infection after antibiotic treatment or motility disturbance. Cell Host Microbe 16, 770–777. doi: 10.1016/j.chom.2014.11.003, PMID: 25498344 PMC4859344

[ref25] Goh Y. J. Klaenhammer T. R. (2015). Genetic mechanisms of prebiotic oligosaccharide metabolism in probiotic microbes. Annu. Rev. Food Sci. Technol. 6, 137–156. doi: 10.1146/annurev-food-022814-015706, PMID: 25532597

[ref26] Goluszko P. Nowicki B. (2005). Membrane cholesterol: a crucial molecule affecting interactions of microbial pathogens with mammalian cells. Infect. Immun. 73, 7791–7796. doi: 10.1128/IAI.73.12.7791-7796.2005, PMID: 16299268 PMC1307024

[ref27] Gong B. Li H. Feng Y. Zeng S. Zhuo Z. Luo J.. (2022). Prevalence, serotype distribution and antimicrobial resistance of non-Typhoidal Salmonella in hospitalized patients in Conghua District of Guangzhou. China. Front Cell Infect Microbiol 12:805384. doi: 10.3389/fcimb.2022.805384, PMID: 35186792 PMC8847451

[ref28] Gu Z. Eils R. Schlesner M. (2016). Complex heatmaps reveal patterns and correlations in multidimensional genomic data. Bioinformatics 32, 2847–2849. doi: 10.1093/bioinformatics/btw313, PMID: 27207943

[ref29] Guo H. Ingolia N. T. Weissman J. S. Bartel D. P. (2010). Mammalian microRNAs predominantly act to decrease target mRNA levels. Nature 466, 835–840. doi: 10.1038/nature09267, PMID: 20703300 PMC2990499

[ref30] Heithoff D. M. Shimp W. R. House J. K. Xie Y. Weimer B. C. Sinsheimer R. L.. (2012). Intraspecies variation in the emergence of hyperinfectious bacterial strains in nature. PLoS Pathog. 8:e1002647. doi: 10.1371/journal.ppat.1002647, PMID: 22511871 PMC3325197

[ref31] Henard C. A. Bourret T. J. Song M. Vázquez-Torres A. (2010). Control of redox balance by the stringent response regulatory protein promotes antioxidant defenses of Salmonella. J. Biol. Chem. 285, 36785–36793. doi: 10.1074/jbc.M110.160960, PMID: 20851888 PMC2978607

[ref32] Hibberd A. A. Yde C. C. Ziegler M. L. Honoré A. H. Saarinen M. T. Lahtinen S.. (2019). Probiotic or synbiotic alters the gut microbiota and metabolism in a randomised controlled trial of weight management in overweight adults. Benef Microbes 10, 121–135. doi: 10.3920/BM2018.0028, PMID: 30525950

[ref33] Hu G. Ling C. Chi L. Thind M. K. Furse S. Koulman A.. (2022). The role of the tryptophan-NAD + pathway in a mouse model of severe malnutrition induced liver dysfunction. Nat. Commun. 13:7576. doi: 10.1038/s41467-022-35317-y, PMID: 36481684 PMC9732354

[ref34] Hume P. J. Singh V. Davidson A. C. Koronakis V. (2017). Swiss Army pathogen: the Salmonella entry toolkit. Front. Cell. Infect. Microbiol. 7:348. doi: 10.3389/fcimb.2017.00348 28848711 PMC5552672

[ref35] Jelsbak L. Hartman H. Schroll C. Rosenkrantz J. T. Lemire S. Wallrodt I.. (2014). Identification of metabolic pathways essential for fitness of *Salmonella Typhimurium* in vivo. PLoS One 9:e101869. doi: 10.1371/journal.pone.0101869, PMID: 24992475 PMC4081726

[ref36] Karp P. D. Billington R. Caspi R. Fulcher C. A. Latendresse M. Kothari A.. (2019). The BioCyc collection of microbial genomes and metabolic pathways. Brief. Bioinform. 20, 1085–1093. doi: 10.1093/bib/bbx085, PMID: 29447345 PMC6781571

[ref37] Kerr A. K. Farrar A. M. Waddell L. A. Wilkins W. Wilhelm B. J. Bucher O.. (2013). A systematic review-meta-analysis and meta-regression on the effect of selected competitive exclusion products on Salmonella spp. prevalence and concentration in broiler chickens. Prev. Vet. Med. 111, 112–125. doi: 10.1016/j.prevetmed.2013.04.005, PMID: 23731553

[ref38] Khan S. Moore R. J. Stanley D. Chousalkar K. K. (2020). The gut microbiota of laying hens and its manipulation with prebiotics and probiotics to enhance gut health and food safety. Appl. Environ. Microbiol. 86:e00600-20. doi: 10.1128/AEM.00600-20 32332137 PMC7301851

[ref39] Kim D. Paggi J. M. Park C. Bennett C. Salzberg S. L. (2019). Graph-based genome alignment and genotyping with HISAT2 and HISAT-genotype. Nat. Biotechnol. 37, 907–915. doi: 10.1038/s41587-019-0201-4, PMID: 31375807 PMC7605509

[ref40] Kim D. Seo S. U. Zeng M. Y. Kim W. U. Kamada N. Inohara N.. (2017). Mesenchymal cell-specific MyD88 signaling promotes systemic dissemination of *Salmonella Typhimurium* via inflammatory monocytes. J. Immunol. 199, 1362–1371. doi: 10.4049/jimmunol.1601527, PMID: 28674182 PMC5548622

[ref41] Kolenda R. Ugorski M. Grzymajlo K. (2019). Everything You always wanted to know about Salmonella type 1 fimbriae, but were afraid to ask. Front. Microbiol. 10:1017. doi: 10.3389/fmicb.2019.01017, PMID: 31139165 PMC6527747

[ref42] Kong N.., Production and analysis of high molecular weight genomic DNA for NGS pipelines using Agilent DNA extraction kit (p/n 200600). Santa Clara, CA, USA: Agilent Technologies. (2013).

[ref43] Langmead B. Salzberg S. L. (2012). Fast gapped-read alignment with bowtie 2. Nat. Methods 9, 357–359. doi: 10.1038/nmeth.1923, PMID: 22388286 PMC3322381

[ref44] Lee J. Y. Tsolis R. M. Baumler A. J. (2022). The microbiome and gut homeostasis. Science 377:eabp9960. doi: 10.1126/science.abp9960 35771903

[ref45] Li H. Handsaker B. Wysoker A. Fennell T. Ruan J. Homer N.. (2009). The sequence alignment/map format and SAMtools. Bioinformatics 25, 2078–2079. doi: 10.1093/bioinformatics/btp352, PMID: 19505943 PMC2723002

[ref46] Liao Y. Smyth G. K. Shi W. (2014). featureCounts: an efficient general purpose program for assigning sequence reads to genomic features. Bioinformatics 30, 923–930. doi: 10.1093/bioinformatics/btt656, PMID: 24227677

[ref47] Liu R. T. Walsh R. F. L. Sheehan A. E. (2019). Prebiotics and probiotics for depression and anxiety: a systematic review and meta-analysis of controlled clinical trials. Neurosci. Biobehav. Rev. 102, 13–23. doi: 10.1016/j.neubiorev.2019.03.023, PMID: 31004628 PMC6584030

[ref48] Maathuis A. J. van den Heuvel E. Schoterman M. H. Venema K. (2012). Galacto-oligosaccharides have prebiotic activity in a dynamic in vitro colon model using a (13) C-labeling technique. J. Nutr. 142, 1205–1212. doi: 10.3945/jn.111.157420, PMID: 22623395

[ref49] Maga E. A. Weimer B. C. Murray J. D. (2013). Dissecting the role of milk components on gut microbiota composition. Gut Microbes 4, 136–139. doi: 10.4161/gmic.23188, PMID: 23235404 PMC3595073

[ref50] Mano M. C. R. Neri-Numa I. A. da Silva J. B. Paulino B. N. Pessoa M. G. Pastore G. M. (2018). Oligosaccharide biotechnology: an approach of prebiotic revolution on the industry. Appl. Microbiol. Biotechnol. 102, 17–37. doi: 10.1007/s00253-017-8564-2, PMID: 29032473

[ref51] Marcobal A. Barboza M. Sonnenburg E. D. Pudlo N. Martens E. C. Desai P.. (2011). Bacteroides in the infant gut consume milk oligosaccharides via mucus-utilization pathways. Cell Host Microbe 10, 507–514. doi: 10.1016/j.chom.2011.10.007, PMID: 22036470 PMC3227561

[ref52] Margolis A. Liu L. Porwollik S. Till J. K. A. Chu W. McClelland M.. (2023). Arginine metabolism powers Salmonella resistance to oxidative stress. Infect. Immun. 91:e0012023. doi: 10.1128/iai.00120-23, PMID: 37191509 PMC10269097

[ref53] McGuckin M. A. Lindén S. K. Sutton P. Florin T. H. (2011). Mucin dynamics and enteric pathogens. Nat. Rev. Microbiol. 9, 265–278. doi: 10.1038/nrmicro2538, PMID: 21407243

[ref54] Mohamad-Fauzi N. Shaw C. Foutouhi S. H. Hess M. Kong N. Kol A.. (2023). Salmonella enhances osteogenic differentiation in adipose-derived mesenchymal stem cells. Front. Cell Dev. Biol.:11. doi: 10.3389/fcell.2023.1077350 PMC10055666 37009487

[ref55] Newman A. M. Arshad M. (2020). The role of probiotics, prebiotics and Synbiotics in combating multidrug-resistant organisms. Clin. Ther. 42, 1637–1648. doi: 10.1016/j.clinthera.2020.06.011, PMID: 32800382 PMC7904027

[ref56] Nguyen B. D. Cuenca V. M. Hartl J. Gül E. Bauer R. Meile S.. (2020). Import of aspartate and malate by DcuABC drives H2/fumarate respiration to promote initial Salmonella gut-lumen colonization in mice. Cell Host Microbe 27, 922–936.e6. doi: 10.1016/j.chom.2020.04.013 32416061 PMC7292772

[ref57] Oba S. Sunagawa T. Tanihiro R. Awashima K. Sugiyama H. Odani T.. (2020). Prebiotic effects of yeast mannan, which selectively promotes Bacteroides thetaiotaomicron and *Bacteroides ovatus* in a human colonic microbiota model. Sci. Rep. 10:17351. doi: 10.1038/s41598-020-74379-0, PMID: 33060635 PMC7562712

[ref58] Pang Z. Zhou G. Ewald J. Chang L. Hacariz O. Basu N.. (2022). Using MetaboAnalyst 5.0 for LC–HRMS spectra processing, multi-omics integration and covariate adjustment of global metabolomics data. Nat. Protoc. 17, 1735–1761. doi: 10.1038/s41596-022-00710-w, PMID: 35715522

[ref59] Park D. Arabyan N. Williams C. C. Song T. Mitra A. Weimer B. C.. (2016). *Salmonella Typhimurium* enzymatically landscapes the host intestinal epithelial cell (IEC) surface Glycome to increase invasion. Mol. Cell. Proteomics 15, 3653–3664. doi: 10.1074/mcp.M116.063206, PMID: 27754876 PMC5141278

[ref60] Perdijk O. van Baarlen P. Fernandez-Gutierrez M. M. van den Brink E. Schuren F. H. J. Brugman S.. (2019). Sialyllactose and Galactooligosaccharides promote epithelial barrier functioning and distinctly modulate microbiota composition and short chain fatty acid production in vitro. Front. Immunol. 10:94. doi: 10.3389/fimmu.2019.00094, PMID: 30809221 PMC6380229

[ref61] Plaza-Diaz J. Fontana L. Gil A. (2018). Human milk oligosaccharides and immune system development. Nutrients 10:1038. doi: 10.3390/nu10081038 30096792 PMC6116142

[ref62] Ren W. Rajendran R. Zhao Y. Tan B. Wu G. Bazer F. W.. (2018). Amino acids as mediators of metabolic cross talk between host and pathogen. Front. Immunol. 9:319. doi: 10.3389/fimmu.2018.00319 29535717 PMC5835074

[ref63] Rentas M. F. Pedreira R. S. Perini M. P. Risolia L. W. Zafalon R. V. A. Alvarenga I. C.. (2020). Galactoligosaccharide and a prebiotic blend improve colonic health and immunity of adult dogs. PLoS One 15:e0238006. doi: 10.1371/journal.pone.0238006, PMID: 32857814 PMC7455039

[ref64] Robinson M. D. McCarthy D. J. Smyth G. K. (2010). edgeR: a Bioconductor package for differential expression analysis of digital gene expression data. Bioinformatics 26, 139–140. doi: 10.1093/bioinformatics/btp616 19910308 PMC2796818

[ref65] Rousseaux A. Brosseau C. le Gall S. Piloquet H. Barbarot S. Bodinier M. (2021). Human Milk oligosaccharides: their effects on the host and their potential as therapeutic agents. Front. Immunol. 12:680911. doi: 10.3389/fimmu.2021.680911, PMID: 34108974 PMC8180913

[ref66] Ruiz-Palacios G. M. Cervantes L. E. Ramos P. Chavez-Munguia B. Newburg D. S. (2003). *Campylobacter jejuni* binds intestinal H(O) antigen (Fuc alpha 1, 2Gal beta 1, 4GlcNAc), and fucosyloligosaccharides of human milk inhibit its binding and infection. J. Biol. Chem. 278, 14112–14120. doi: 10.1074/jbc.M207744200, PMID: 12562767

[ref67] Rychlik I. Barrow P. A. (2005). Salmonella stress management and its relevance to behaviour during intestinal colonisation and infection. FEMS Microbiol. Rev. 29, 1021–1040. doi: 10.1016/j.femsre.2005.03.005 16023758

[ref68] Shah J. Desai P. T. Chen D. Stevens J. R. Weimer B. C. (2013). Preadaptation to cold stress in *Salmonella enterica* serovar Typhimurium increases survival during subsequent acid stress exposure. Appl. Environ. Microbiol. 79, 7281–7289. doi: 10.1128/AEM.02621-13, PMID: 24056458 PMC3837722

[ref69] Shah J. Desai P. T. Weimer B. C. (2014). Genetic mechanisms underlying the pathogenicity of cold-stressed *Salmonella enterica* serovar typhimurium in cultured intestinal epithelial cells. Appl. Environ. Microbiol. 80, 6943–6953. doi: 10.1128/AEM.01994-14, PMID: 25192993 PMC4249018

[ref70] Shaw C. Hess M. Weimer B. C. (2023). Microbial-derived tryptophan metabolites and their role in neurological disease: anthranilic acid and anthranilic acid derivatives. Microorganisms 11:1825. doi: 10.3390/microorganisms11071825 37512997 PMC10384668

[ref71] Shaw C. Weimer B. C. Gann R. Desai P. T. Shah J. D. (2024). The Yin and Yang of pathogens and probiotics: interplay between *Salmonella enterica* sv. Typhimurium and *Bifidobacterium infantis* during co-infection. Front. Microbiol. 15:1387498. doi: 10.3389/fmicb.2024.1387498, PMID: 38812689 PMC11133690

[ref72] Shaw C. A. Verstrate M. Graniczkowska K. Risoen K. R. Dini P. Weimer B. C. (2025). The use of stem cells and organoids for modeling host-microbe interactions in low-biomass tissues. Front. Cell. Infect. Microbiol. 15:1641366. doi: 10.3389/fcimb.2025.1641366 40909341 PMC12405370

[ref73] Shoaf K. Mulvey G. L. Armstrong G. D. Hutkins R. W. (2006). Prebiotic galactooligosaccharides reduce adherence of enteropathogenic *Escherichia coli* to tissue culture cells. Infect. Immun. 74, 6920–6928. doi: 10.1128/IAI.01030-06, PMID: 16982832 PMC1698067

[ref74] Spiga L. Winter M. G. Furtado de Carvalho T. Zhu W. Hughes E. R. Gillis C. C.. (2017). An oxidative central metabolism enables Salmonella to utilize microbiota-derived succinate. Cell Host Microbe 22, 291–301.e6. doi: 10.1016/j.chom.2017.07.018, PMID: 28844888 PMC5599368

[ref75] Spring P. Wenk C. Connolly A. Kiers A. (2015). A review of 733 published trials on bio-Mos®, a mannan oligosaccharide, and Actigen®, a second generation mannose rich fraction, on farm and companion animals. J. Appl. Anim. Nutr. 3:e8. doi: 10.1017/jan.2015.6

[ref76] Strum J. S. Aldredge D. Barile D. Lebrilla C. B. (2012). Coupling flash liquid chromatography with mass spectrometry for enrichment and isolation of milk oligosaccharides for functional studies. Anal. Biochem. 424, 87–96. doi: 10.1016/j.ab.2012.02.012, PMID: 22370281 PMC3562133

[ref77] Tingirikari J. M. R. (2018). Microbiota-accessible pectic poly- and oligosaccharides in gut health. Food Funct. 9, 5059–5073. doi: 10.1039/c8fo01296b, PMID: 30280147

[ref78] Unden G. Steinmetz P. A. Degreif-Dunnwald P. (2014). The aerobic and anaerobic respiratory chain of Escherichia coli and *Salmonella enterica*: enzymes and energetics. EcoSal Plus 6. doi: 10.1128/ecosalplus.esp-0005-2013, PMID: 26442941

[ref79] Velez E. Castillo N. Mesón O. Grau A. Bibas Bonet M. E. Perdigón G. (2013). Study of the effect exerted by fructo-oligosaccharides from yacon (Smallanthus sonchifolius) root flour in an intestinal infection model with *Salmonella Typhimurium*. Br. J. Nutr. 109, 1971–1979. doi: 10.1017/S0007114512004230, PMID: 23137694

[ref80] Wahlig T. A. Bixler B. J. Valdés-López O. Mysore K. S. Wen J. Ané J. M.. (2019). Salmonella enterica serovar Typhimurium ATCC 14028S is tolerant to plant defenses triggered by the flagellin receptor FLS2. FEMS Microbiol. Lett. 366:fny296. doi: 10.1093/femsle/fny296, PMID: 30601977 PMC6420342

[ref81] Wang K. C.. (2014). FimY of *Salmonella enterica* serovar Typhimurium functions as a DNA-binding protein and binds the fimZ promoter. Microbiol. Res. 169, 496–503. doi: 10.1016/j.micres.2013.12.006 24462182

[ref82] Yeung A. T. Y. Choi Y. H. Lee A. H. Y. Hale C. Ponstingl H. Pickard D.. (2019). A genome-wide knockout screen in human macrophages identified host factors modulating Salmonella infection. MBio 10:e02169-19. doi: 10.1128/mBio.02169-19, PMID: 31594818 PMC6786873

[ref83] You S. Ma Y. Yan B. Pei W. Wu Q. Ding C.. (2022). The promotion mechanism of prebiotics for probiotics: a review. Front. Nutr. 9:1000517. doi: 10.3389/fnut.2022.1000517, PMID: 36276830 PMC9581195

[ref84] Zhan R. Han Q. Zhang C. Tian Z. Zhang J. (2015). Toll-like receptor 2 (TLR2) and TLR9 play opposing roles in host innate immunity against *Salmonella enterica* serovar Typhimurium infection. Infect. Immun. 83, 1641–1649. doi: 10.1128/IAI.02870-14, PMID: 25667264 PMC4363406

[ref85] Zhu X. Guo Y. Liu Z. Yang J. Tang H. Wang Y. (2021). Itaconic acid exerts anti-inflammatory and antibacterial effects via promoting pentose phosphate pathway to produce ROS. Sci. Rep. 11:18173. doi: 10.1038/s41598-021-97352-x, PMID: 34518559 PMC8438069

