# Supplementary material for: Cellular respiration and amino acid metabolism is altered by dietary oligosaccharides in Salmonella with epithelial cell association
Source: Front Microbiol. 2025 Oct 7;16:1672770. doi: 10.3389/fmicb.2025.1672770 (PMC12537660; doi:10.3389/fmicb.2025.1672770)
Supplement: Supplementary file 1 [file Data_Sheet_1.docx]

**Supplemental Figures**

***Supplemental Figure 1.* Expression of a subset of transmembrane receptors in Caco2 cells diverges by prebiotic treatment.** Transmembrane receptor expression was measured in Caco2 cells pretreated with either HMO or BioMos then infected. Genes were clustered by Euclidian Distance and expression data is log_2_ fold change with non-prebiotic treated by infected Caco2 cells.

***Supplemental Figure 2.* Metabolic profiles of prebiotic treatments from *S.* Typhimurium 14028s infected Caco2 cells cluster by prebiotic type.** K-Means clustering, with cluster setting of 2, of metabolomes from *S.* Typhimurium infection across time and between prebiotic treatment groups across 60 min *S.* Typhimurium co-incubation. T_0_ STM v T_60_ STM compares metabolites from Caco2/STM treatment across 0min and 60min time points. BioMos vs HMO compares metabolites across prebiotic pre-treatments at time 0 mins and 60 mins.

***Supplemental Figure 3.* Metabolic profiles of all conditions reveal distinct differences driven by both prebiotic treatment and *S.* Typhimurium 14028s presence/absence.** Correlation plot of untargeted metabolic profiles of all treatment combinations and individual replicates A-D for each combination created using MetaboAnalyst. Pink to red squares indicate positive correlation between metabolic profiles whereas gradients of blue represent negative correlations between profiles across sample type. Colored bars on the right-hand side of the plot indicate sample grouping by prebiotic type, pathogen presence/absence, and time course. Time 0 corresponds to 15 minutes post prebiotic addition and initiation of *S.* Typhimurium addition. Time 60 is 60 minutes post *S.* Typhimurium inclusion or 75 minutes after initial prebiotic addition when a *S.* Typhimurium is not added.

***Supplemental Figure 4.* Metabolites are significantly altered by prebiotic treatment and infection status.** The metabolic profiles underlying the correlation plot in Supplemental Figure 4 were used to search for significance across all treatment combinations. The Kruskal Wallis Test in MetaboAnalyst revealed 252 significant metabolites out of 316 total.

***Supplemental Figure 5.* Graphical abstract of basic experimental set-up.** Caco2 cells (ATCC HTB-37) were grown and differentiated before being pre-treated with 1% HMO or BioMos for 15 mins. Stationary *S. enterica* sv. Typhimurium 14028 was then added to the cells and co-incubated for 60 mins. Cells and supernatant were collected, washed and stored for RNA-seq and metabolomics follow-up.

**
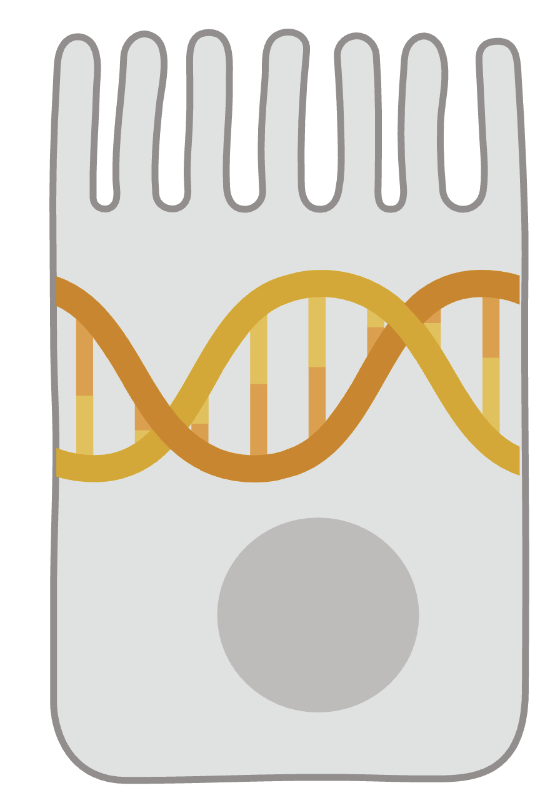
**

**
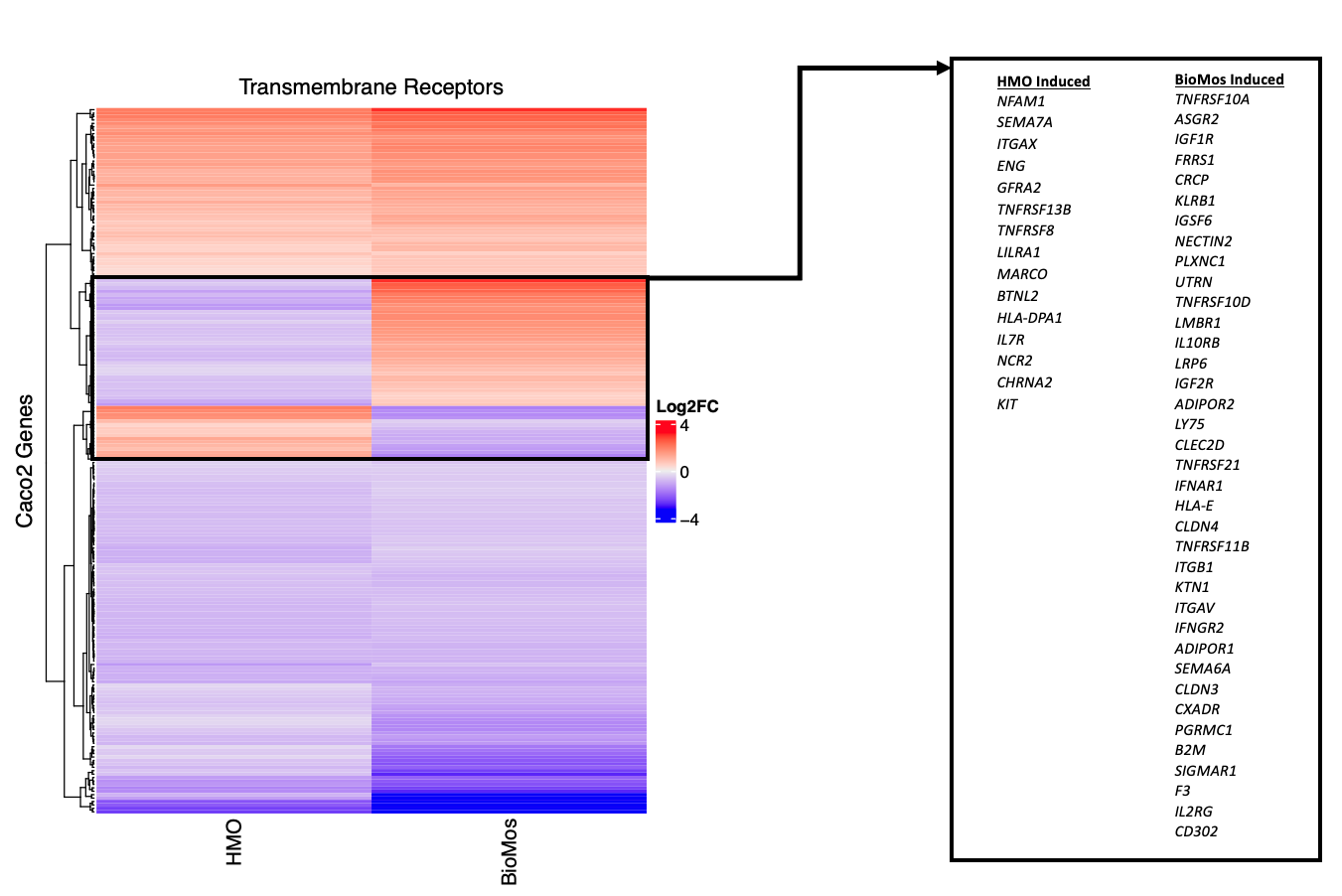
**

**Supplemental Figure 1.**

**Supplemental Figure 2.**

**Supplemental Figure 3.**

**Supplemental Figure 4.**


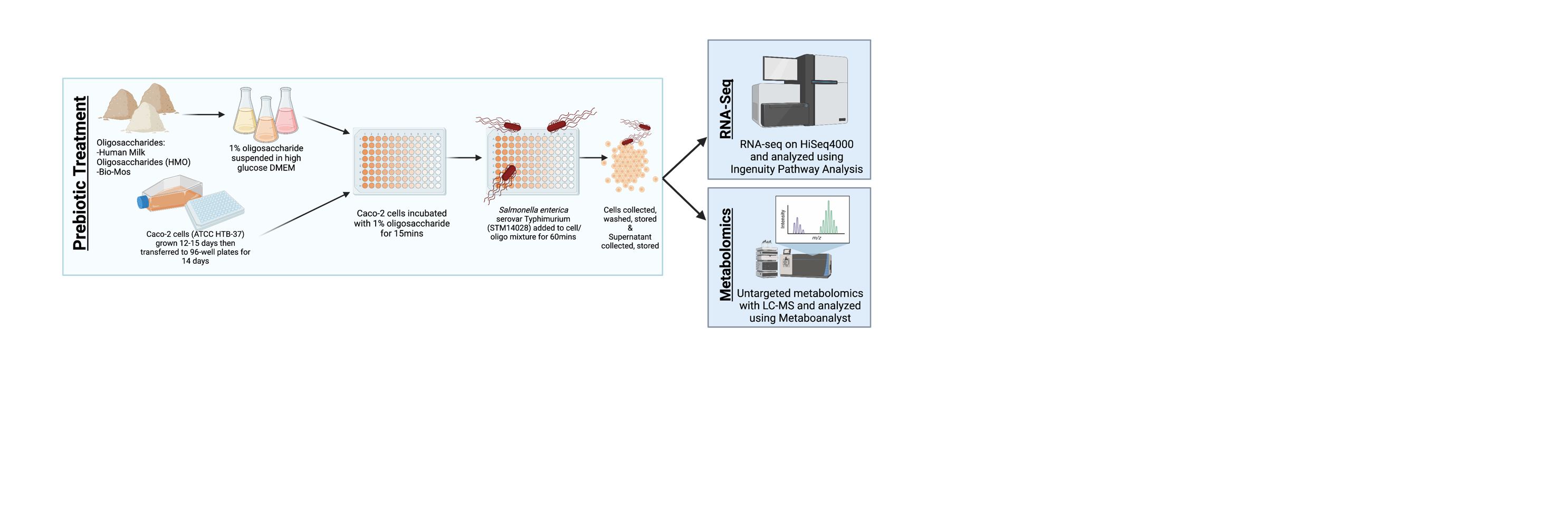


**Supplemental Figure 5.**
